# Morphological and molecular analyses reveal two new insular species of *Cnemaspis* Strauch, 1887 (Squamata, Gekkonidae) from Satun Province, southern Thailand

**DOI:** 10.3897/zookeys.858.34297

**Published:** 2019-07-01

**Authors:** Natee Ampai, Attapol Rujirawan, Perry L. Wood Jr, Bryan L. Stuart, Anchalee Aowphol

**Affiliations:** 1 Department of Zoology, Faculty of Science, Kasetsart University, Bangkok, 10900 Thailand Kasetsart University Bangkok Thailand; 2 Department of Biological Sciences and Museum of Natural History, Auburn University, Auburn, AL, USA Auburn University Auburn United States of America; 3 Section of Research and Collections, North Carolina Museum of Natural Sciences, Raleigh, NC, USA North Carolina Museum of Natural Sciences Raleigh United States of America

**Keywords:** Island, rock geckos, species diversity, systematics

## Abstract

We describe two new insular gecko species of the genus *Cnemaspis* from Tarutao, Adang, and Rawi islands in Satun Province, southern Thailand. The new species are distinguished from their congeners in having a unique combination of morphological, scalation, and color pattern characters, and by genetic divergence in the mitochondrial NADH dehydrogenase subunit 2 (ND2) gene. *Cnemaspistarutaoensis***sp. nov.** was found to be a member of the *C.kumpoli* group, but is distinguished from all other species in that group by having 8–9 supralabials and 8 infralabials; 4–5 pore-bearing precloacal scales, pores rounded; 17–19 paravertebral tubercles randomly arranged; 27–29 subdigital lamellae under the fourth toe; subcaudal region yellowish, with smooth scales and a single enlarged median row; black gular markings in males and females; and 17.24–22.36% uncorrected pairwise sequence divergences. *Cnemaspisadangrawi***sp. nov.** was found to be a member of the *C.siamensis* group, but is distinguished from all other species in that group by having 10 supralabials and 9 infralabials; 6–8 pore-bearing precloacal scales, pores rounded and arranged in a chevron shape; 23–25 randomly arranged, separated paravertebral tubercle rows; 26–28 subdigital lamellae under the fourth toe; subcaudal scales keeled, without enlarged median row; gular region, abdomen, limbs and subcaudal region yellowish in males only; gular marking absent in males and females; and 8.30–26.38 % uncorrected pairwise sequence divergences. *Cnemaspistarutaoensis***sp. nov.** occurs in karst formations on Tarutao Island, while *Cnemaspisadangrawi***sp. nov.** is found near granitic, rocky streams on Adang and Rawi islands.

## Introduction

Southeast Asia is a global biodiversity hotspot with extraordinary levels of species endemism (Myers et al. 2000). Southern Thailand serves as an important biogeographic transition zone between the Indochinese and Sundaic biotas, especially at the Isthmus of Kra and the Kangar-pattani line (Hughes et al. 2003; Woodruff and Turner 2009; Woodruff 2010; Parnell 2013). Southern Thailand has high levels of species diversity and endemism of reptiles (Sodhi et al. 2004; Grismer et al. 2010; Das and van Dijk 2013; Wood et al. 2017).

The rock gecko genus *Cnemaspis* Strauch, 1887 currently contains 57 recognized species distributed throughout Southeast Asia (Grismer et al. 2014; Riyanto et al. 2017; Wood et al. 2017; Uetz et al. 2018). The number of recognized *Cnemaspis* species has increased rapidly during the past two decades (e.g. Bauer and Das 1998; Das 2005; Bauer et al. 2007; Grismer et al. 2009, 2014, 2015b; Grismer and Chan 2010; Wood et al. 2013, 2017; Riyanto et al. 2017). Thailand currently contains 16 recognized species of *Cnemaspis* (Grismer et al. 2010, 2014; Wood et al. 2017; Uetz et al. 2018) ranging from Chanthaburi in the east (Bauer and Das 1998), Sai Yok to the west (Grismer et al. 2010), and south through the Thai peninsula to the Malaysian border and its offshore islands (Grismer et al. 2014; Wood et al. 2017). Species delimitation of *Cnemaspis* in Thailand has been hindered by their conserved morphology and microhabitat specialization (Bauer and Das 1998; Grismer et al. 2010, 2014; Wood et al. 2017). Earlier taxonomic studies on *Cnemaspis* relied on morphology (e.g. Smith 1925; Taylor 1963; Bauer and Das 1998; Das and Leong 2004) but recent studies have incorporated molecular data to aid clarifying species boundaries in Thailand (e.g. Grismer et al. 2010; 2014; Wood et al. 2017). Grismer et al. (2014) recognized four groups of *Cnemaspis* in Thailand on the basis of morphological and molecular data: the *siamensis* group, the *chanthaburiensis* group, the *kumpoli* group (= Pattani clade of Grismer et al. 2014), and the *affinis* group. The *siamensis* group contains species that occur throughout western Thailand, southward to southern Thailand, and include *C.chanardi* Grismer, Sumontha, Cota, Grismer, Wood, Pauwels & Kunya, *C.huaseesom* Grismer, Sumontha, Cota, Grismer, Wood, Pauwels & Kunya, *C.kamolnoranathi* Grismer, Sumontha, Cota, Grismer, Wood, Pauwels & Kunya, *C.omari* Grismer, Wood, Anuar, Riyanto, Ahmad, Muin, Sumontha, Grismer, Onn, Quah & Pauwels, *C.phangngaensis* Wood, Grismer, Aowphol, Aguilar, Cota, Grismer, Murdoch & Sites, *C.punctatonuchalis* Grismer, Sumontha, Cota, Grismer, Wood, Pauwels & Kunya, *C.roticanai* Grismer & Onn, *C.siamensis* Smith, *C.thachanaensis* Wood, Grismer, Aowphol, Aguilar, Cota, Grismer, Murdoch & Sites, and *C.vandeventeri* Grismer, Sumontha, Cota, Grismer, Wood, Pauwels & Kunya. The *chanthaburiensis* group contains species that occur from the northern margin of the Gulf of Thailand, eastward to Cambodia and southern Vietnam, and include *C.aurantiacopes* Grismer & Ngo, *C.caudanivea* Grismer & Ngo, *C.chanthaburiensis* Bauer & Das, *C.lineogularis* Wood, Grismer, Aowphol, Aguilar, Cota, Grismer, Murdoch & Sites, *C.neangthyi* Grismer, Grismer & Chav, *C.nuicamensis* Grismer & Ngo, and *C.tucdupensis* Grismer & Ngo. The *kumpoli* group is composed of four species, *C.biocellata* Grismer, Chan, Nasir & Sumontha, *C.kumpoli* Taylor, *C.monachorum* Grismer, Ahmad, Chan, Belabut, Muin, Wood & Grismer, and *C.niyomwanae* Grismer, Sumontha, Cota, Grismer, Wood, Pauwels & Kunya, that occur from southern Thailand to northern Malaysia. The *affinis* group contains species that occur from southern Thailand to central Peninsular Malaysia, including *C.affinis* Stoliczka, *C.harimau* Chan, Grismer, Anuar, Quah, Muin, Savage, Grismer, Ahmad, Remigio & Greer, *C.pseudomcguirei* Grismer, Ahmad, Chan, Belabut, Muin, Wood, Grismer, *C.shahruli* Grismer, Chan, Quah, Muin Savage, Grismer, Ahmad, Greer & Remegio, *C.mcguirei* Grismer, Grismer, Wood & Chan, *C.grismeri* Wood, Quah, Anuar & Muin, *C.flavolineata* Nicholls, *C.temiah* Grismer, Wood, Anuar, Riyanto, Ahmad, Muin, Sumontha, Grismer, Onn, Quah & Pauwels, *C.narathiwatensis* Grismer, Sumontha, Cota, Grismer, Wood, Pauwels & Kunya, *C.hangus* Grismer, Wood, Anuar, Riyanto, Ahmad, Muin, Sumontha, Grismer, Onn, Quah & Pauwels, *C.selamatkanmerapoh* Grismer, Wood, Mohamed, Chan, Heinz, Sumarli, Chan & Loredo, *C.bayuensis* Grismer, Grismer, Wood & Chan, and *C.stongensis* Grismer, Wood, Anuar, Riyanto, Ahmad, Muin, Sumontha, Grismer, Onn, Quah & Pauwels. Although a large number of *Cnemaspis* species have been reported from Thailand, only two species are known to occur on islands in Thailand: *C.chanardi* from Samui, Phangan and Ko Tao islands, and *C.siamensis* from Phuket Island (Grismer et al. 2010, 2014). It is evident that the diversity of *Cnemaspis* on the islands of southern Thailand, especially those containing isolated karst formations and granitic rocky streams, remains poorly studied.

During recent fieldwork in 2017–2018 on Tarutao, Adang, and Rawi islands, Satun Province, southern Thailand, specimens of *Cnemaspis* were collected that differed from all other named species. Herein, we evaluate the morphological and molecular distinctiveness of these specimens.

## Materials and methods

### Sampling

**Figure 1.**
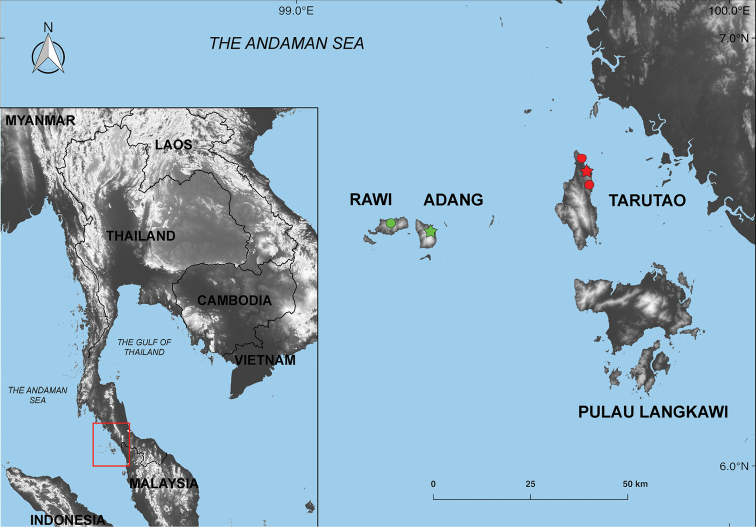
Map illustrating the holotype locality (red star) and paratype localities (red circles) of *Cnemaspistarutaoensis* sp. nov. at Tarutao Island, Satun Province, Thailand; the holotype locality (green star) and paratype localities (green circles) of *Cnemaspisadangrawi* sp. nov. at Adang and Rawi islands, Mueang Satun District, Satun Province, Thailand.

Specimens of *Cnemaspis* were collected from Tarutao, Adang, and Rawi islands in Tarutao National Park, Mueang Satun District, Satun Province, Thailand (Fig. 1) between November 2017–April 2018. Specimens were collected by hand during the day (1000–1800 h) and at night (1900–2200 h). Liver or muscle samples for genetic analysis were collected and preserved in 95% ethanol after euthanasia. Specimens were fixed in 10% formalin and later transferred to 70% ethanol for permanent storage. Specimens and tissue samples were deposited in the herpetological collection at the Zoological Museum of Kasetsart University, Bangkok, Thailand (**ZMKU**) and the Thailand Natural History Museum, Pathum Thani, Thailand (**THNHM**).

### Morphology

Only adult individuals were used in the morphological analysis, as determined by the presence of hemipenes or precloacal pores in males, and the presence of calcium glands or eggs in females. Measurements were taken by the first author on the left side of preserved specimens to the nearest 0.1 mm using digital calipers under a Nikon SMZ 445 dissecting microscope. Sixteen measurements were taken following Grismer et al. (2014) and Wood et al. (2017): snout-vent length (**SVL**), taken from tip of snout to the anterior margin of vent; tail width (**TW**) at the base of the tail immediately posterior to the postcloacal swelling; tail length (**TL**), as distance from the vent to the tip of the tail, whether original or regenerated; forearm length (**FL**), taken on the dorsal surface from the posterior margin of the elbow while flexed 90° to the inflection of the flexed wrist; tibia length (**TBL**), taken on the ventral surface from the posterior surface of the knee while flexed 90° to the base of the heel; head length (**HL**), as distance from the posterior margin of the retroarticular process of the lower jaw to the tip of the snout; head width (**HW**) at the angle of the jaws; head depth (**HD**), as the maximum height of head from the occiput to the throat; axilla-groin length (**AG**), taken from the posterior margin of the forelimb at its insertion point on the body to the anterior margin of the hind limb at its insertion point on the body; eye diameter (**ED**), as the maximum horizontal diameter of the eyeball; eye-snout distance (**ES**), measured from the anterior margin of the eyeball to the tip of snout; eye-ear distance (**EE**), measured from the anterior edge of the ear opening to the posterior edge of the eyeball; eye-nostril distance (**EN**), measured from the anterior most margin of the eyeball to the posterior margin of the external nares; inner orbital distance (**IO**), as the width of the frontal bone at the level of the anterior edges of the orbit; internarial distance (**IN**), measured between the medial margins of the nares across the rostrum; and ear length (**EL**), taken from the greatest vertical distance of the ear opening.

Meristic characters of scale counts and external observations of morphology were taken following Grismer et al. (2014) and Wood et al. (2017): number of supralabial and infralabial scales, counted from below the middle of the orbit to the rostral and mental scales, respectively; texture of scales on the anterior margin of the forearm; number of paravertebral tubercles between limb insertions, counted in a straight line immediately left of the vertebral column; presence or absence of a row of enlarged, widely spaced, tubercles along the ventrolateral edge of the body flank between limb insertions; number of subdigital lamellae beneath the fourth toe (=4^th^ toe lamellae), counted from the base of the first phalanx to the claw; general size (i.e., strong, moderate, weak) and arrangement (i.e., random or linear) of dorsal body tubercles; number, orientation and shape of precloacal pores; relative size of subcaudal and subtibial scales; and number of postcloacal tubercles on each side of tail base.

Comparative material was examined in the holdings of **THNHM** (Appendix 1), and comparative data were obtained from the original descriptions of other Thai species of *Cnemaspis* (Grismer et al. 2009; Grismer and Chan 2010; Grismer et al. 2010; Wood et al. 2017).

### Molecules

Genomic DNA was extracted from liver tissue of eight individuals of *Cnemaspis* (Table 1) using the Qiagen DNAeasy tissue kit (Valencia, CA, USA). An 1,296 bp fragment of mitochondrial (mt) DNA consisting of the NADH dehydrogenase subunit 2 (ND2) gene and the flanking tRNAs Trp, Ala, Asn and Cys was amplified by the polymerase chain reaction (PCR; 95 °C for 2 min, 95 °C for 35 s, 52 °C for 35s, 72 °C for 35 s) for 33 cycles using the primers L4437b (5’-AAGCAGTTGGGCCCATACC-3’; Macey et al. 1997) and H5934 (5’ AGRGTGCCAATGTCTTTGTGRTT-3’; Macey et al. 1997). PCR products were purified using the AccuPrep® PCR Purification Kit (Bioneer, Daejeon, Korea), and were sequenced using the amplifying primers and the internal sequencing primer CyrtintF1 (5’-TAGCCYTCTCYTCYATYGCCC-3’; Siler et al. 2010) on an ABI 3730 automatic sequencer (Applied Biosystems, CA, USA). Sequences were edited and aligned using Geneious v.5.6.3 (Biomatters, Auckland, New Zealand). All new sequences were deposited in GenBank under accession numbers MK862112 to MK862119 (Table 1).

**Table 1.** Samples used in this study, including catalogue numbers, Genbank accession numbers and localities of voucher specimens. Voucher abbreviations are as follows: Monte L. Bean Life Science Museum at Brigham Young University (**BYU**), California Academy of Sciences (**CAS**), the Field Museum of Natural History, Chicago, Illinois, USA (**FMNH**), La Sierra University Herpetological Collection (**LSUHC**), Universiti Sains Malaysia Herpetological Collection at the Universiti Sains Malaysia, Penang, Malaysia (**USMHC**), and Zoological Museum of Kasetsart University (**ZMKU**).

Species	Locality	Collection no.	Genbank accession no.	Reference
*Cyrtodactylusintermedius*	Cambodia, Kampot	FMNH 263228	KT13107	Grismer et al. 2015a
*Hemidactylusgarnotii*	Myanmar, Mon State, Kyaihto Township, Kyait Hti Yo Wildlife Sanctuary.	CAS 222276	EU68364	Bauer et al. 2008
*Cnemaspisadangrawi* sp. nov.	Thailand, Satun Province, Mueang Satun District, Adang Island	ZMKU R 00767	MK862112	This study
	Thailand, Satun Province, Mueang Satun District, Adang Island	THNHM 28207	MK862113	This study
	Thailand, Satun Province, Mueang Satun District, Adang Island	ZMKU R 00770	MK862114	This study
	Thailand, Satun Province, Mueang Satun District, Rawi Island	ZMKU R 00775	MK862115	This study
	Thailand, Satun Province, Mueang Satun District, Rawi Island	ZMKU R 00776	MK862116	This study
*Cnemaspisaffinis*	Malaysia, Penang, Pulau Pinang	LSUHC 6787	KM024682	Grismer et al. 2014
*Cnemaspisargus*	Malaysia, Terengganu, Gunung Lawit	LSUHC 8304	KM024687	Grismer et al. 2014
	Malaysia, Terengganu, Gunung Lawit	LSUHC 10834	KM024688	Grismer et al. 2014
*Cnemaspisaurantiacopes*	Vietnam, Kien Giang Province, Hon Dat Hill	LSUHC 8610	KM024692	Grismer et al. 2014
	Vietnam, Kien Giang Province, Hon Dat Hill	LSUHC 8611	KM024693	Grismer et al. 2014
*Cnemaspisbiocellata*	Malaysia, Perlis, Kuala Perlis	LSUHC 8817	KM024707	Grismer et al. 2014
	Malaysia, Perlis, Kuala Perlis	LSUHC 8817	KM024708	Grismer et al. 2014
	Malaysia, Perlis, Gua Kelam	LSUHC 8789	KM024709	Grismer et al. 2014
*Cnemaspisboulengerii*	Vietnam, Ca Mau Province, Con Dao Archipelago	LSUHC9278	KM024710	Grismer et al. 2014
	Vietnam, Ca Mau Province, Con Dao Archipelago	LSUHC9279	KM024711	Grismer et al. 2014
*Cnemaspiscaudanivea*	Vietnam, Kien Giang Province, Hon Tre Island	LSUHC 8582	KM024714	Grismer et al. 2014
*Cnemaspischanardi*	Thailand, Nakhon Si Thammarat Province, Thum Thong Panra	LSUHC 9567	KM024715	Grismer et al. 2014
*Cnemaspischanthaburiensis*	Cambodia, Pursat Province, Phnom Dalai	LSUHC 9338	KM024716	Grismer et al. 2014
*Cnemaspisgrismeri*	Malaysia, Perak, Lenggong	LSUHC 9969	KM024722	Grismer et al. 2014
*Cnemaspishangus*	Malaysia, Pahang, Bukit Hangus	LSUHC 9358b	KM024728	Grismer et al. 2014
*Cnemaspisharimau*	Malaysia, Kedah, Gunung Jeri	LSUHC 9665	KM024730	Grismer et al. 2014
*Cnemaspishuaseesom*	Thailand, Kanchanaburi Province, Sai Yok National Park	LSUHC 9455	KM024733	Grismer et al. 2014
	Thailand, Kanchanaburi Province, Sai Yok National Park	LSUHC 9457	KM024734	Grismer et al. 2014
	Thailand, Kanchanaburi Province, Sai Yok National Park	LSUHC 9458	KM024735	Grismer et al. 2014
*Cnemaspiskarsticola*	Malaysia, Kelantan, Gunung Reng	LSUHC 9054	KM024736	Grismer et al. 2014
	Malaysia, Kelantan, Gunung Reng	LSUHC 9055	KM024737	Grismer et al. 2014
*Cnemaspiskumpoli*	Malaysia, Perlis, Perlis State Park	LSUHC 8847	KM024745	Grismer et al. 2014
	Malaysia, Perlis, Perlis State Park	LSUHC 8848	KM024746	Grismer et al. 2014
*Cnemaspislineogularis*	Thailand, Prachuap Khiri Khan Province, Kui Buri District, Wat Khao Daeng	BYU 62535	KY091231	Wood et al. 2017
	Thailand, Prachuap Khiri Khan Province, Kui Buri District, Wat Khao Daeng	ZMKU R 00728	KY091233	Wood et al. 2017
*Cnemaspismahsuriae*	Malaysia, Kedah, Pulau Langkawi, Gunung Raya	LSUHC 11829	KT250634	Grismer et al. 2015b
*Cnemaspismcguirei*	Malaysia, Perak, Bukit Larut	LSUHC 8853	KM024751	Grismer et al. 2014
*Cnemaspismonachorum*	Malaysia, Kedah, Langkawi Archipelago, Pulau Langkawi	LSUHC 9114	KM024754	Grismer et al. 2014
	Malaysia, Kedah, Langkawi Archipelago, Pulau Langkawi	LSUHC 10807	KM024755	Grismer et al. 2014
*Cnemaspisnarathiwatensis*	Malaysia, Perak, Belum-Temengor, Sungai Enam	USMHC 1347	KM024762	Grismer et al. 2014
	Malaysia, Perak, Belum-Temengor, Sungai Enam	USMHC 1348	KM024763	Grismer et al. 2014
*Cnemaspisneangthyi*	Cambodia, Pursat Province, O’Lakmeas	LSUHC 8515	KM024767	Grismer et al. 2014
	Cambodia, Pursat Province, O’Lakmeas	LSUHC 8516	KM024768	Grismer et al. 2014
*Cnemaspisniyomwanae*	Thailand, Trang Province, Thum Khao Ting	LSUHC 9568	KM024773	Grismer et al. 2014
	Thailand, Trang Province, Thum Khao Ting	LSUHC 9571	KM024774	Grismer et al. 2014
*Cnemaspisnuicamensis*	Vietnam, An Giang Province, Nui Cam Hill	LSUHC 8646	KM024775	Grismer et al. 2014
	Vietnam, An Giang Province, Nui Cam Hill	LSUHC 8647	KM024776	Grismer et al. 2014
	Vietnam, An Giang Province, Nui Cam Hill	LSUHC 8648	KM024777	Grismer et al. 2014
*Cnemaspisomari*	Thailand, Satun Province, Phuphaphet Cave	LSUHC 9565	KM024780	Grismer et al. 2014
	Malaysia, Perlis, Perlis State Park	LSUHC 9978	KM024779	Grismer et al. 2014
*Cnemaspisperhentianensis*	Malaysia, Terengganu, Pulau Perhentian Besar	LSUHC 8699	KM024820	Grismer et al. 2014
*Cnemaspisphangngaensis*	Thailand, Phangnga Province, Mueang Phangnga District, Khao Chang, Phung Chang Cave	BYU 62537	KY091234	Wood et al. 2017
	Thailand, Phangnga Province, Mueang Phangnga District, Khao Chang, Phung Chang Cave	BYU 62538	KY091235	Wood et al. 2017
*Cnemaspispunctatonuchalis*	Thailand, Prachaup Khiri Khan Province, Thap Sakae	BYU 62539	KY091236	Wood et al. 2017
	Thailand, Prachaup Khiri Khan Province, Thap Sakae	BYU 62540	KY091237	Wood et al. 2017
*Cnemaspisroticanai*	Malaysia, Kedah, Pulau Langkawi, Gunung Raya	LSUHC 9430	KM024829	Grismer et al. 2014
	Malaysia, Kedah, Pulau Langkawi, Gunung Raya	LSUHC 9431	KM024830	Grismer et al. 2014
	Malaysia, Kedah, Pulau Langkawi, Gunung Raya	LSUHC 9439	KM024831	Grismer et al. 2014
*Cnemaspissiamensis*	Thailand, Chumpon Province, Pathio District	LSUHC 9474	KM024838	Grismer et al. 2014
	Thailand, Chumpon Province, Pathio District	LSUHC 9485	KM024839	Grismer et al. 2014
*Cnemaspistarutaoensis* sp. nov.	Thailand, Satun Province, Mueang Satun District, Tarutao Island	ZMKU R 00761	MK862117	This study
	Thailand, Satun Province, Mueang Satun District, Tarutao Island	ZMKUR 00763	MK862118	This study
	Thailand, Satun Province, Mueang Satun District, Tarutao Island	ZMKU R 00764	MK862119	This study
*Cnemaspisthachanaensis*	Thailand, Surat Thani Province, Tha Chana District, Tham Khao Sonk Hill	BYU 62542	KY091239	Wood et al. 2017
	Thailand, Surat Thani Province, Tha Chana District, Tham Khao Sonk Hill	BYU 62543	KY091243	Wood et al. 2017
	Thailand, Surat Thani Province, Tha Chana District, Tham Khao Sonk Hill	BYU 62544	KY091244	Wood et al. 2017
*Cnemaspistucdupensis*	Vietnam, An Giang Province, Tuc Dup Hill	LSUHC 8631	KM024852	Grismer et al. 2014
	Vietnam, An Giang Province, Tuc Dup Hill	LSUHC 8632	KM024853	Grismer et al. 2014
*Cnemaspisvandeventeri*	Thailand, Ranong Province, Suk Saran District, Naka	BYU 62541	KY091238	Wood et al. 2017

### Phylogenetic analyses

Homologous sequences of 56 *Cnemaspis* and the outgroups *Cyrtodactylusintermedius* and *Hemidactylusgarnotii* (following Bauer et al. 2008; Grismer et al. 2015b) were downloaded from GenBank and aligned to the eight newly generated *Cnemaspis* sequences using Geneious v. 5.6.3 (Biomatters, Auckland, New Zealand). The aligned dataset was partitioned into four partitions consisting of ND2 first, second and third codon positions, and tRNAs.

Phylogenies were reconstructed with the maximum likelihood (ML) criterion using IQ-TREE v. 1.6.7 (Nguyen et al. 2014) on the IQ-TREE web server (Trifinopoulos et al. 2016). The best-fit model of substitution for each partition was estimated using IQ-TREE’s ModelFinder function (Kalyaanamoorthy et al. 2017) under the Akaike Information Criterion (AIC). The selected models were TIM+F+R4 for each ND2 codon position partition, and HKY+F+R4 for the tRNA partition. Bootstrap analysis was performed using the ultrafast bootstrap approximation (Minh et al. 2013) with 1,000 replicates and 0.95 minimum correlation coefficient.

Phylogenies were also reconstructed with Bayesian Inference (BI) using MrBayes v. 3.2 on XSEDE on the Cyberinfrastructure for Phylogenetic Research (CIPRES; Miller et al. 2010) computer cluster. The best-fit model of substitution was estimated for each partition using jModelTest 2.1.10 (Posada 2008) under AIC. The selected models were GTR+ I+Γ for each ND2 codon position partition, and HKY+ I+Γ for the tRNA partition. Two simultaneous runs, each with three heated and one cold chain, were performed using the default priors for 10 × 10^6^ generations, with trees sampled every 1,000 generations from the Markov Chain Monte Carlo (MCMC). Runs were halted after the average standard deviation of split frequencies was below 0.01 and convergence was assumed. The first 25% of the trees were discarded as burn-in using the sumt command. The convergence of the two simultaneous runs, and stationary state of each parameter, were evaluated using Tracer v. 1.6 (Rambaut et al. 2014). Runs were terminated when the effective sample sizes (ESS) of all parameters was greater than or equal to 200.

The most likely tree in the ML analysis, and the 50% majority-rule consensus of the sampled trees from the BI analysis, were visualized using FigTree v. 1.4.3 (Rambaut 2009). Nodes having bootstrap support (BS) of ≥70 and posterior probabilities (PP) of ≥0.95 were considered to be well-supported (Huelsenbeck and Ronquist 2001; Wilcox et al. 2002). Uncorrected pairwise sequence divergences were calculated using MEGA v. 7.0.26 (Kumar et al. 2016).

## Results

### Molecular analyses

The aligned dataset contained 1,296 characters of 64 individuals of *Cnemaspis* and two individuals of the outgroup species. The standard deviation of split frequencies among the two simultaneous BI runs was 0.001478. The ESS values were greater than or equal to 3,630 for all parameters. A single most likely tree resulted from the ML analysis.

**Figure 2.**
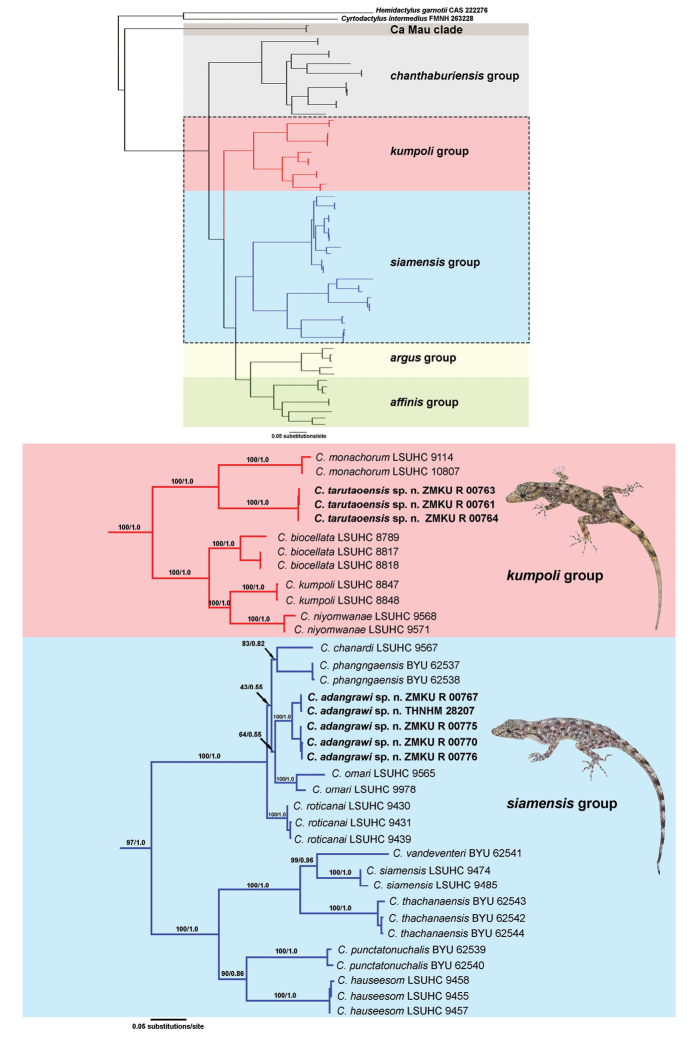
The single best maximum likelihood tree of the mitochondrial NADH dehydrogenase subunit 2 (ND2) gene and flanking tRNAs from geckos of the genera *Cnemaspis*, *Cyrtodactylus* and *Hemidactylus*, shown in full view (above) and close-up view of relevant clades (below). Support values at nodes are bootstrap values from a maximum likelihood analysis of the same dataset followed by posterior probabilities of the Bayesian analysis.

The most likely ML tree and the 50% majority rule consensus tree from the BI analysis had similar topologies (Fig. 2). *Cnemaspis* samples from Tarutao Island represented a well-supported lineage (100 BS, 1.0 PP) within the *kumpoli* group, and was recovered as the sister species to *C.monachorum* from Pulau Langkawi, Malaysia (100 BS, 1.0 PP). The Tarutao samples differed from one another by uncorrected *p*-distances of 0.00–0.31%, but from other members of the *kumpoli* group by uncorrected *p*-distances of 17.24–22.36 % (Table 2).

**Table 2.** Mean (minimum–maximum) uncorrected *p*-distances (%) within the *Cnemaspiskumpoli* group based on 1,296 bp of the mitochondrial ND2 gene and flanking tRNAs. Numbers in bold are within species divergence. *n* = number of individuals.

No.	Species	*n*	1	2	3	4	5
1	*C.tarutaoensis* sp. n.	3	**0.16 (0.00–0.31)**				
2	*C.monachorum*	2	17.70 (17.24–18.17)	**0.54 (0.00–1.09)**			
3	*C.biocellata*	3	20.34 (20.19–20.50)	19.10 (19.79–19.41)	**3.57 (0.00–7.14)**		
4	*C.kumpoli*	2	21.84 (21.74–21.89)	22.28 (22.95–22.52)	13.51 (13.51–13.51)	**0.16 (0.00–0.31)**	
5	*C.niyomwanae*	2	21.35 (20.34–22.36)	21.20 (19.88–22.36)	14.44 (13.20–15.68)	12.89 (11.49–14.29)	**1.79 (0.00–3.57)**

*Cnemaspis* samples from Adang and Rawi islands represented a well-supported lineage (100 BS, 1.0 PP) within the *siamensis* group, and was recovered as being closely related to a clade containing *C.chanardi*, *C.phangngaensis*, *C.omari*, and *C.roticanai* (Fig. 2). However, the exact sister taxon relationship of the Adang and Rawi islands was not resolved with strong support (Fig. 2). The Adang-Rawi samples differed from one another by uncorrected *p*-distances of 0.00–4.68 %, but from other members of the *siamensis* group by uncorrected *p*-distances of 8.30–26.38 % (Table 3).

**Table 3.** Mean (minimum-maximum) uncorrected *p*-distances (%) within the *Cnemaspsissiamensis* group based on 1,296 bp of the mitochondrial ND2 gene and flanking tRNAs. Numbers in bold are within species divergence. *n* = number of individuals.

No.	Species	*n*	1	2	3	4	5	6	7	8	9	10
1	*C.adangrawi* sp. nov.	5	**2.81**									
			**(0.00–4.68)**									
2	*C.chanardi*	1	11.40	**0.00**								
			(10.85–11.91)									
3	*C.omari*	2	9.36	11.81	**2.13**							
			(8.30–10.21)	(11.49–12.13)	**(0.00–4.26)**							
4	*C.phangngaensis*	2	10.19	11.38	11.17	**0.11**						
			(9.57–10.85)	(11.27–11.49)	(10.85–11.49)	**(0.00–0.21)**						
5	*C.siamensis*	2	25.83	24.40	27.77	25.00	**0.00**					
			(25.74–25.96)	(24.26–24.68)	(27.66–27.87)	(24.89–25.11)						
6	*C.roticanai*	3	8.92	11.77	9.01	8.90	28.16	**0.11**				
			(8.51–9.57)	(11.70–11.91)	(8.72–9.36)	(8.72–9.15)	(28.09–28.30)	**(0.00–0.21)**				
7	*C.vandeventeri*	1	24.26	24.04	26.60	25.21	12.34	26.88	**0.00**			
			(24.04–24.47)	(24.04–24.04)	(25.96–27.23)	(25.11–25.32)	(12.34–12.34)	(26.81–27.02)				
8	*C.thachanaensis*	3	25.50	24.40	28.30	26.13	13.35	27.66	14.47	**0.53**		
			(25.10–25.96)	(24.26–24.68)	(27.23–28.94)	(25.74–26.81)	(13.19–14.26)	(27.45–28.09)	(14.26–14.89)	**(0.00–1.06)**		
9	*C.punctatonuchalis*	2	25.23	25.53	26.38	25.00	19.36	25.60	21.06	21.13	**0.00**	
			(24.04–26.17)	(25.53–25.53)	(26.38–26.38)	(25.00–25.00)	(19.36–19.36)	(25.53–25.74)	(21.06–21.06)	(21.06–21.28)		
10	*C.huaseesom*	3	26.00	26.17	28.19	23.72	19.36	27.52	20.64	20.99	16.95	**0.43**
			(25.74–26.38)	(26.17–26.17)	(27.87–28.51)	(23.62–23.83)	(19.36–19.36)	(27.45–27.66)	(20.64–20.64)	(20.64–21.70)	(16.81–17.02)	**(0.00–0.64)**

### Taxonomic hypotheses

The Tarutao and Adang-Rawi samples of *Cnemaspis* differed from each other and all other congeners by being diagnosable in morphology and mitochondrial DNA. Owing to these independent lines of evidence, we hypothesize that the Tarutao and Adang-Rawi samples represent two new species to science, and are described below.

### Systematics

#### Cnemaspis tarutaoensis sp. nov.

Taxon classificationAnimaliaSquamataGekkonidae

http://zoobank.org/91BAE519-9241-447C-8EB6-BED473B99529

**Figure 3.**
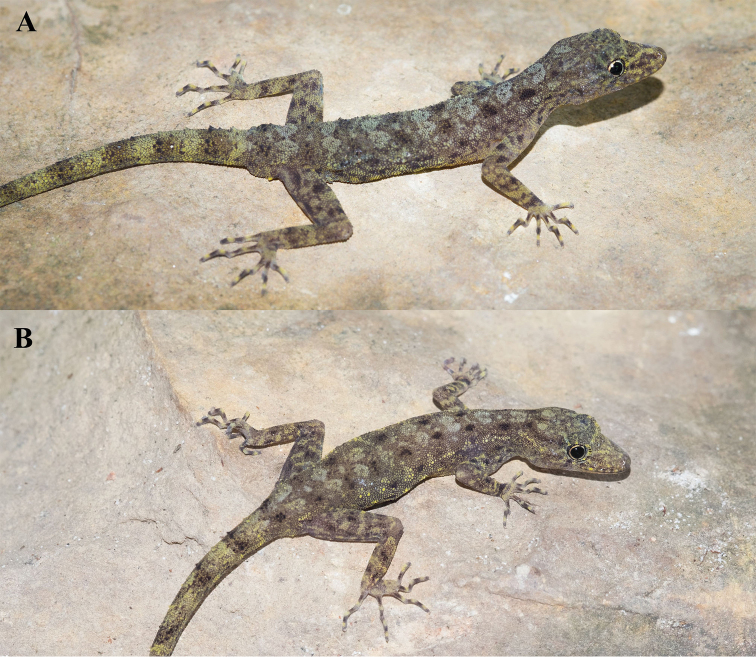
*Cnemaspistarutaoensis* sp. nov. from Tarutao Island, Mueang Satun District, Satun Province, Thailand. **A** male holotype ZMKU R 00763 **B** female paratype ZMKU R 00758.

**Figure 4.**
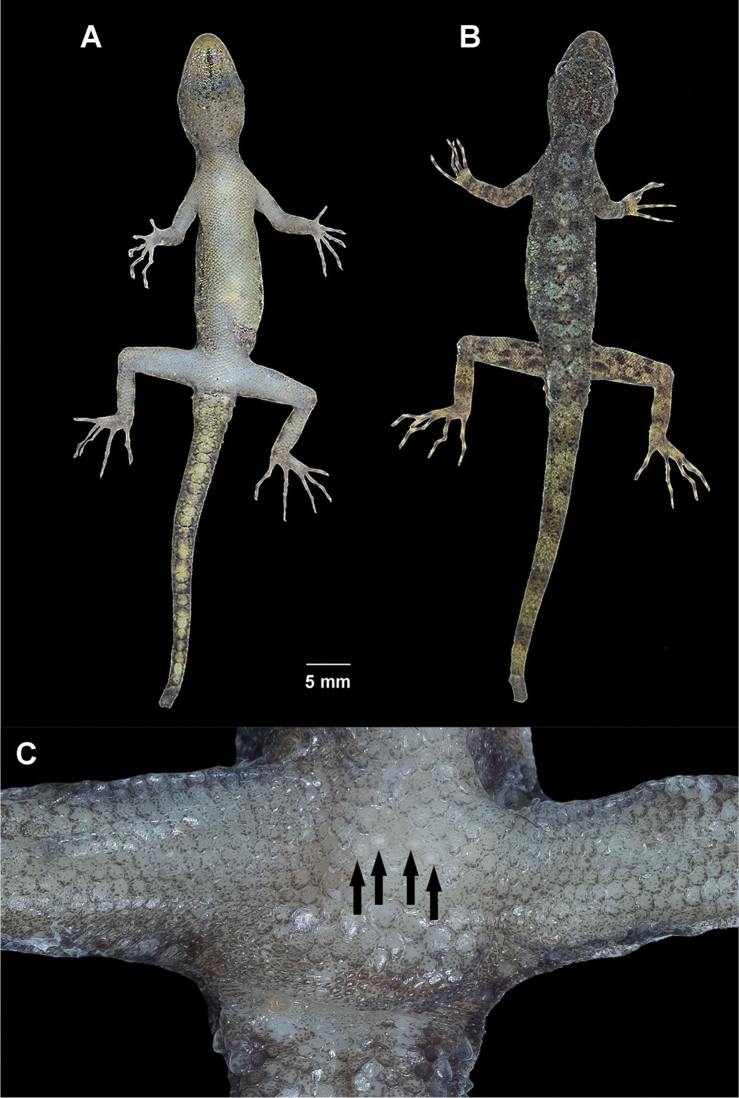
Male holotype of *Cnemaspistarutaoensis* sp. nov. from Tarutao Island, Mueang Satun District, Satun Province, Thailand (ZMKU R 00763) in life. **A** ventral view **B** dorsal view **C** precloacal region showing distribution of pore-bearing scales (black arrows).

**Figure 5.**
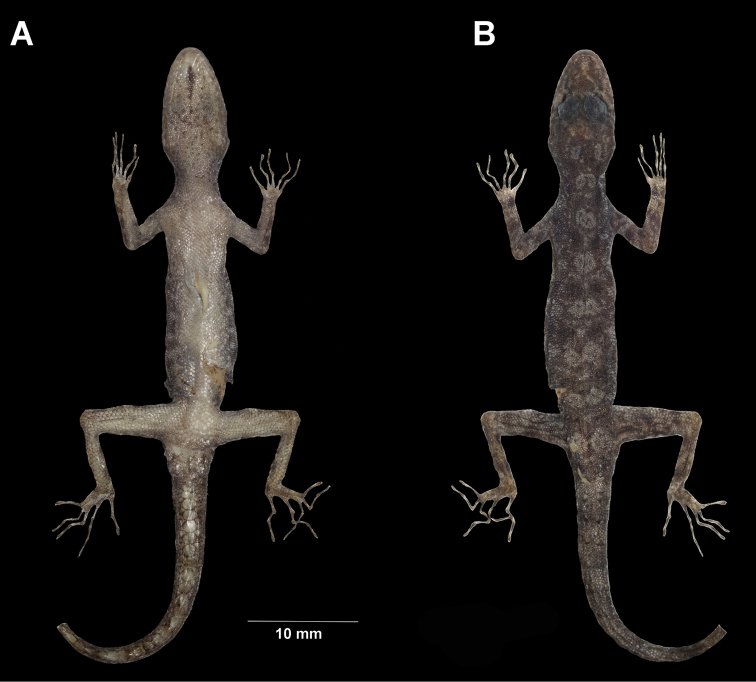
Male holotype of *Cnemaspistarutaoensis* sp. nov. from Tarutao Island, Mueang Satun District, Satun Province, Thailand (ZMKU R 00763) in preservative. **A** ventral **B** dorsal views.

**Figure 6.**
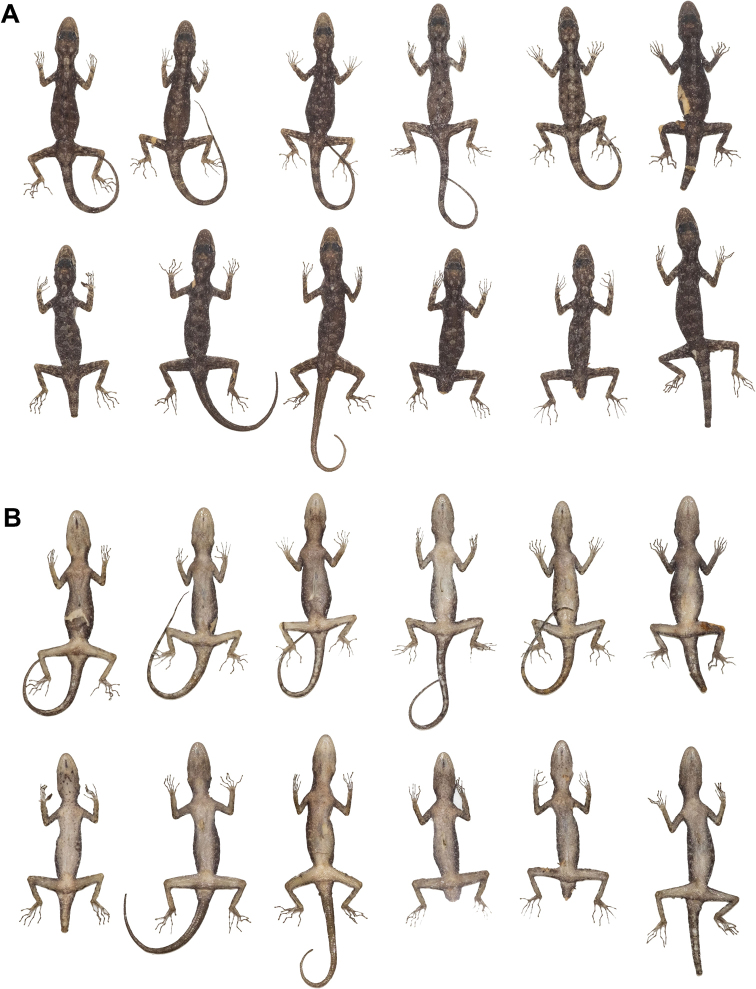
Paratypes of *Cnemaspistarutaoensis* sp. nov. in preservative. **A** dorsal view **B** ventral view; from left to right, top panel (females): ZMKU R 00758, ZMKU R 00759, ZMKU R 00760, ZMKU R 00766, THNHM 28201, and THNHM 28202; bottom panel (males): ZMKU R 00761, ZMKU R 00762, ZMKU R 00765, ZMKU R 00764, THNHM 28203, and THNHM 28205.

Figures 3 , 4 , 5 , 6

##### Holotype

**Figure 7.**
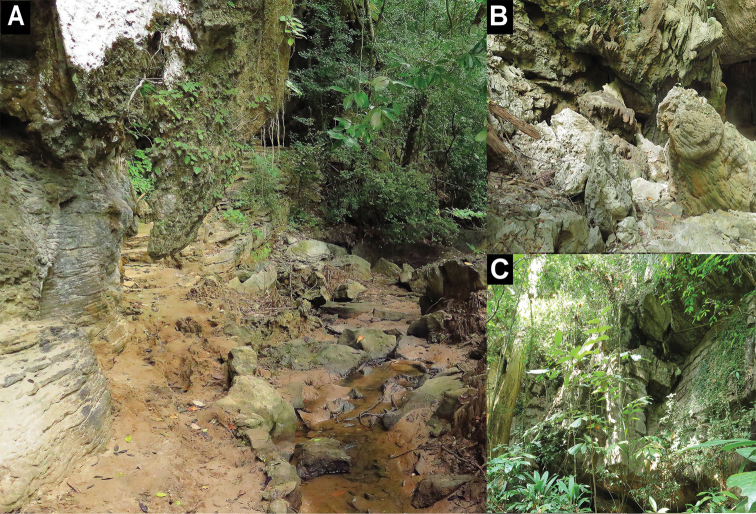
Habitats of *Cnemaspistarutaoensis* sp. nov. **A** Pha Toe Boo karst formation at type locality **B** habitat of paratypes in the exterior surface of karst cave at Tham Chorakae **C** habitat of paratypes in karst outcropped at Tarutao Island, Mueang Satun district, Satun Province, Thailand.

(Figs 3A, 4, 5). ZMKU R 00763, adult male from Thailand, Satun Province, Mueang Satun District, Tarutao National Park, Tarutao Island, Pha Toe Boo (6°42.1854'N, 99°38.8956'E; 2 m a.s.l.; Fig. 7A), collected on 5 November 2017 by Natee Ampai, Attapol Rujirawan, Siriporn Yodthong, and Korkwan Termprayoon.

##### Paratypes

(Figs 3b, 6). Twelve paratypes (adult males = 6, adult females = 6). ZMKU R 00761–00762, ZMKU R 00764 (3 adult males), THNHM 28201–28202, ZMKU R 00758–00760 (5 adult females), bear the same collection data as holotype. THNHM 28203 (1 adult male), same data as holotype except collected 5 April 2018. ZMKU R 00765 (1 adult male), same data as holotype except collected at Tham Chorakhae (6°41.7966'N, 99°39.0426'E; 37 m a.s.l.; Fig. 7B), collected 7 November 2017. ZMKU R 00766 (1 adult female) and THNHM 28205 (1 adult male), same data as holotype except collected at karst forest near stream (6°39.759'N, 99°39.1596'E; 53 m a.s.l.; Fig. 7C), collected 5 April 2018.

##### Referred specimens.

THNHM 28204 (one juvenile), same data as holotype except collected 5 April 2018.

##### Diagnosis.

*Cnemaspistarutaoensis* sp. nov. can be distinguished from all other *Cnemaspis* by having the following combination of characters: (1) adult males with maximum snout-vent length (SVL) 36.4 mm (mean 34.7 ± SD 1.5, *n* = 7) and females with maximum SVL 34.8 mm (mean 33.7 ± SD 0.6, *n* = 6); (2) 8–9 supralabials and 8 infralabials; (3) 4–5 pore-bearing precloacal scales, pores rounded; (4) 17–19 paravertebral tubercles, small in size, randomly arranged; (5) 27–29 subdigital lamellae under the 4^th^ toe; (6) subcaudal region yellowish, scales smooth with a single enlarged median subcaudal row; (7) one postcloacal tubercles on each side; (8) no sexual dimorphism in dorsal and ventral patterns; and (9) black gular markings present in males and females. These differences are summarized for geographically close congeners in the *kumpoli* group (Table 4).

**Table 4.** Meristic character states and color patterns of species in the *Cnemaspiskumpoli* group. Measurements are taken in millimeters and measurement abbreviations are defined in the text. var = character variable; – = data unavailable, ant = anterior.

Characters/Species	*C.tarutaoensis* sp. nov.	*C.biocellata*	*C.kumpoli*	*C.monachorum*	*C.niyomwanae*
Sample size	13	25	13	12	5
Maximum SVL	36.4	40.2	63.0	32.9	56.8
Supralabial scales	8–9	6–10	7–9	7–8	8–11
Infralabial scales	8	5–9	6–8	5–7	6–8
Ventral scales keeled (1) or smooth (0)	0	0	0	0	0
No. of precloacal pores	4–5	6–12	1–8	3	3
Precloacal pore continuous (1) or separated (0)	0.1	1	0	1	0.1
No. of paravertebral tubercles	17–19	21–27	28–35	11–20	26–31
Tubercles present (1) or absent (0) on lower flanks	0	1	1	0	0
No. of 4^th^ toe lamellae	27–29	29–37	34–41	24–30	31–34
Lateral caudal furrows present (1) or absent (0)	1	1	1	1	1
Lateral caudal tubercle row present (1) or absent (0)	0	ant	0	ant	0
Subcaudal scales keeled (1) or smooth (0)	0	0	0	0	0
Enlarge submetatarsal scales on 1^st^ toe (1) or not (0)	0	0	0	0	0
Enlarge median subcaudal scales row (1) or not (0)	1	1	1	1	1
No. of postcloacal tubercles in males	1	1	2.3	1–2	1.2
Subcaudal region yellow present (1) or not (0)	1	var	0	0	0
Ventral pattern sexually dimorphic present (1) or not (0)	0	1	0	1	–
Dorsal color pattern sexually dimorphic (1) or not (0)	0	1	1	0	1
Wide black and yellow bands on tail present (1) or not (0)	1	0	0	0	0
Gular marking (1) or not (0)	1	0	0	1	0

##### Description of holotype.

Adult male; SVL 36.3 mm; head moderate in size (HL/SVL 0.28), elongate, narrow (HW/SVL 0.15), flattened (HD/HL 0.33), distinct from neck; snout moderate (ES/HL 0.42), in lateral view slightly concave; postnasal region constricted medially; scales of rostrum, raised, smooth, larger than conical scales on occiput; faint supraorbital ridges; gular and throat scales raised, smooth and round; shallow frontorostral sulcus; canthus rostralis nearly absent, smoothly rounded; eye large (ED/HL 0.18); pupil round; ear opening oval, taller than wide; rostral slightly concave, dorsal 80% divided by longitudinal median groove; rostral bordered posteriorly by supranasal and laterally by first supralabial; 9, 9 (right, left) slightly raised supralabials decreasing in size posteriorly; 8, 8 (right, left) infralabials decreasing in size posteriorly; nostril elliptical, oriented posterodorsally, bordered posteriorly by small, granular postnasal scales; mental large, triangular, bordered posteriorly by three large postmentals.

Body slender, elongate (AG/SVL 0.39); small, raised and equal in sized, dorsal scales throughout body intermixed with several large, multicarinate tubercles randomly arranged; 19 paravertebral tubercles; tubercles absent on lower flanks; tubercles extend from occiput to base of tail; dorsal scales slightly raised and keeled; pectoral and abdominal scales smooth and round, flat to concave, slightly larger than dorsal scales and not larger posteriorly; ventral scales of brachia raised, smooth and juxtaposed; four pores-bearing precloacal scales arranged in a chevron, separated; precloacal pore rounded; precloacal depression absent; femoral pores absent.

Fore and hind limbs moderately long, slender; scales beneath forearm slightly raised, smooth and subimbricate; subtibial scales keeled; palmar scales smooth and juxtaposed; digits elongate, slender, inflected joint and bearing slightly recurved claws; subdigital lamellae unnotched; lamellae beneath first phalanges wide; lamellae beneath phalanx immediately following inflection granular; lamellae of distal phalanges wide; lamellae beneath inflection large; interdigital webbing absent; enlarge submetatarsal scales on 1^st^ toe absent; fingers increase in length from first to fourth with fourth and fifth nearly equal in length; relative length of fingers IV>V>III>II>I; toes increase in length from first to fifth with fourth and fifth nearly equal in length; relative length of toes IV>V>III>II>I; total number of subdigital lamellae on 4^th^ toe 28, 28 (right, left).

Caudal and subcaudal scales smooth, similar to dorsal scale size; lateral caudal furrow present; lateral caudal tubercle row absent; enlarge caudal tubercles at the base of tail not encircling tail; enlarged median subcaudal scales row present; tail length (TL) 34.3 mm with broken at tail tip; enlarged postcloacal tubercle 1, 1 (right, left) on lateral surface of hemipenial swellings at the base of tail.

##### Coloration in life

(Figs 3, 4). Dorsal ground color of head light brown; top of the head bearing small black, sage and yellowish marking; snout yellowish; dorsal ground color of body, limbs and tail light brown with dark brown to black irregular blotches; ground color of ventral surfaces grayish white intermixed with light yellowish blotches; gular and throat regions are beige and light yellow; anterior gular region yellowish; midgular region with faint, dark lineate marking; thin, faint black postorbital stripe; light sage vertebral blotches extending from the nape to tail; flanks with irregular incomplete sage to yellowish blotches becoming smaller posteriorly; limbs yellowish brown with dark brown incomplete irregular spots subcaudal region yellowish; wide dark brown to black and yellow bands on tail.

##### Coloration in preservative

(Fig. 5). Color pattern similar to that in life with some fading. Dorsal ground color of head, body, limbs and tail brown with vertebral blotches indistinct; irregular pale marking; top of head with indistinct darker marking; all yellow markings faded to whitish gray; dorsal surfaces of limbs with irregular light and dark blotches; entire ventral surface whitish gray; gular region with faint dark lineate marking.

##### Variation.

Most paratypes approximate the holotype in general aspects of color pattern (Fig. 6), with most differences found in the degree of vertebral blotches. ZMKU R 00761 (adult male) has dark spots in gular region. ZMKU R 00762 and ZMKU R 00765 (two adult males) have lighter gular markings than the holotype. THNHM 28201 and ZMKU R 00760 (two adult females) have lighter dorsal markings than the holotype. ZMKU R 00762 and THNHM 28205 (two adult males) have a pattern that resembles transverse bands rather than paravertebral blotches. ZMKU R 00762 and ZMKU R 00765 (two adult males) have regenerated tails of uniform tan coloration. THNHM 28202 (adult female) and THNHM 28203 and THNHM 28205 (two adult males) have broken tails. THNHM 28205 (adult male) is an adult male with five continuous precloacal pores. Meristic and mensural variation within the type series are presented in Table 5.

**Table 5.** Descriptive measurements in millimeters and characters of the type series of *Cnemaspistarutaoensis* sp. nov. M = male; F = female; – = data unavailable or absent; b = broken; r = regenerated.

Museum number	ZMKU	ZMKU	ZMKU	ZMKU	ZMKU	THNHM	THNHM	ZMKU	ZMKU	THNHM	ZMKU	ZMKU	THNHM
	R 00763	R 00761	R 00762	R 00764	R 00765	28203	28205	R 00766	R 00758	28201	R 00759	R 00760	28202
Type series	Holotype	Paratype	Paratype	Paratype	Paratype	Paratype	Paratype	Paratype	Paratype	Paratype	Paratype	Paratype	Paratype
Sex	M	M	M	M	M	M	M	F	F	F	F	F	F
SVL	36.3	33.3	35.2	32.6	35.2	33.8	36.4	33.3	34.8	33.4	33.5	33.8	33.6
TL	34.3b	8.7b	42.3r	b	40.8r	b	17.3b	47.7	45.3	50.3	52.4	44.1r	13.6b
TW	3.5	3.4	3.5	3.2	3.5	3.2	3.6	3.5	3.8	3.5	3.4	3.4	3.3
FL	5.3	5.1	5.2	5.1	5.3	5.1	5.3	5.2	5.2	5.0	5.1	5.1	5.1
TBL	6.3	6.2	6.3	6.0	6.3	6.2	6.3	6.0	6.3	6.0	6.1	6.2	6.1
AG	14.3	14.1	14.3	14.1	14.3	14.1	14.3	14.3	14.2	14.0	14.1	14.0	14.0
HL	10.0	9.8	10.1	9.7	10.0	9.6	10.1	9.5	9.9	9.7	9.7	9.7	9.9
HW	5.6	5.4	5.5	5.4	5.6	5.3	5.6	5.5	5.6	5.2	5.3	5.4	5.3
HD	3.3	3.1	3.2	3.2	3.3	3.2	3.3	3.2	3.2	3.0	3.1	3.2	3.0
ED	1.8	1.9	1.9	1.8	1.8	1.8	1.9	1.8	1.8	1.8	1.9	1.9	1.9
EE	2.9	2.9	3.0	2.9	3.0	2.9	3.0	2.8	2.8	2.7	2.8	2.8	2.8
ES	4.2	4.1	4.1	4.0	4.2	4.0	4.2	4.1	4.2	4.0	4.1	4.0	4.0
EN	3.5	3.2	3.4	3.2	3.5	3.2	3.6	3.4	3.4	3.2	3.2	3.3	3.2
IO	2.2	2.1	2.2	2.0	2.1	2.0	2.2	1.9	2.2	2.1	2.1	2.1	2.0
EL	0.6	0.7	0.7	0.6	0.7	0.6	0.7	0.6	0.7	0.7	0.7	0.6	0.6
IN	0.8	0.7	0.8	0.7	0.8	0.8	0.8	0.7	0.7	0.7	0.8	0.8	0.7
Supralabials	9	9	8	9	9	9	9	9	8	8	8	8	9
Infralabials	8	8	8	8	8	8	8	8	8	8	8	8	8
No. of precloacal pores	4	4	4	4	4	4	5	–	–	–	–	–	–
Precloacal pore continuous (1) or separated (0)	0	0	0	0	0	0	1	–	–	–	–	–	–
No. of paravertebral tubercles	19	19	18	18	19	18	19	17	18	19	18	19	18
No. of 4th toe lamellae	28	29	29	29	29	29	28	29	27	27	27	27	28
Gular marking (1) or absent (0)	1	1	1	1	1	1	1	1	1	1	1	1	1

##### Distribution and natural history.

*Cnemaspistarutaoensis* sp. nov. is known only from the type locality on Tarutao Island, approximately 40 km off the coast of Thailand. All specimens were found in karst forest near mangroves and karst outcrops near a stream (Fig. 7). Nine specimens (ZMKU R 00759–00760, ZMKU R 00762–00763, ZMKU R 00765–00766, and THNHM 28202–28204) were collected during the day (1100–1805 h) and five specimens (ZMKU R 00758, ZMKU R 00761, ZMKU R 00764, THNHM 28201 and THNHM 28205) were collected during the night (1920–2106 h). The male holotype was found during the day (1724 h) upside down on the interior surface of the karst formation.

Paratypes found during the day (ZMKU R 00759 and 00760, ZMKU R 00762 and 00763, ZMKU R 00765–00766, and THNHM 28202–28204) were in shaded areas, cracks, and crevices of rock boulders. When disturbed, some individuals would retreat into cracks and crevices, or hide in shaded areas of the rock boulder. Paratypes found at night (ZMKU R 00758, ZMKU R 00761, ZMKU R 00764, THNHM 28201 and THNHM 28205) were in deep crevices, within cracks on the shaded (by day) surfaces of boulders, or perched on vegetation near karst. Three gravid females (ZMKU R 00758, ZMKU R 00760, and THNHM 28202) contained two eggs during November 2017. THNHM 28204 (juvenile) was observed on vegetation near a rock boulder on 5 April 2018. At night, Cyrtodactyluscf.astrum was found in syntopy on rock boulders and karst formations with *C.tarutaoensis* sp. nov.

##### Etymology.

The specific epithet refers to the type locality of the new species.

##### Comparisons.

*Cnemaspistarutaoensis* sp. nov. can be distinguished from all other members of the *kumpoli* group (*C.biocellata*, *C.kumpoli*, *C.monachorum*, and *C.niyomwanae*) by having a maximum SVL of 36.4 mm (vs 32.9 mm in *C.monachorum*, 40.2 mm in *C.biocellata*, 63.0 mm in *C.kumpoli*, and 56.8 mm in *C.niyomwanae*).

*Cnemaspistarutaoensis* sp. nov. is further distinguished from *C.monachorum* by having eight infralabial scales (vs 5–7 in *C.monachorum*). The new species is further distinguished from *C.biocellata, C.monachorum* and *C.niyomwanae* by having 4–5 precloacal pores (vs 6–12 in *C.biocellata* and three in *C.monachorum* and *C.niyomwanae*). The new species is further distinguished from *C.biocellata*, *C.kumpoli*, and *C.niyomwanae* by having 17–19 paravertebral tubercles (vs 21–27 in *C.biocellata*, 28–35 in *C.kumpoli* and 26–31 in *C.niyomwanae*). The new species is further distinguished from *C.biocellata* and *C.kumpoli* by lacking tubercles on lower flanks (vs present in *C.biocellata* and *C.kumpoli*). The new species is further distinguished from *C.biocellata*, *C.kumpoli* and *C.niyomwanae* by having 26–29 lamellae under the 4^th^ toe (vs 29–37 in *C.biocellata*, 34–41 in *C.kumpoli*, and 31–34 in *C.niyomwanae*).

*Cnemaspistarutaoensis* sp. nov. is further distinguished from *C.kumpoli*, *C.monachorum* and *C.niyomwanae* by having yellow coloration in the subcaudal region and wide black and yellow bands on tail (vs lacking in *C.kumpoli*, *C.monachorum*, and *C.niyomwanae*). The new species is further distinguished from *C.biocellata*, *C.kumpoli*, and *C.niyomwanae* by lacking a sexually dimorphic dorsal color pattern (vs present in *C.biocellata*, *C.kumpoli*, and *C.niyomwanae*). The new species is further distinguished from *C.monachorum* and *C.biocellata* by lacking lateral caudal tubercle row (vs present in *C.monachorum* and *C.biocellata*). The new species is distinguished from *C.biocellata*, *C.kumpoli*, and *C.niyomwanae* by having gular marking (vs lacking in *C.biocellata*, *C.kumpoli*, and *C.niyomwanae*).

#### Cnemaspis adangrawi sp. nov.

Taxon classificationAnimaliaSquamataGekkonidae

http://zoobank.org/E783766E-6BA0-4F3D-A1BD-968C130AB52B

Figures 8 , 9 , 10

##### Holotype

**Figure 8.**
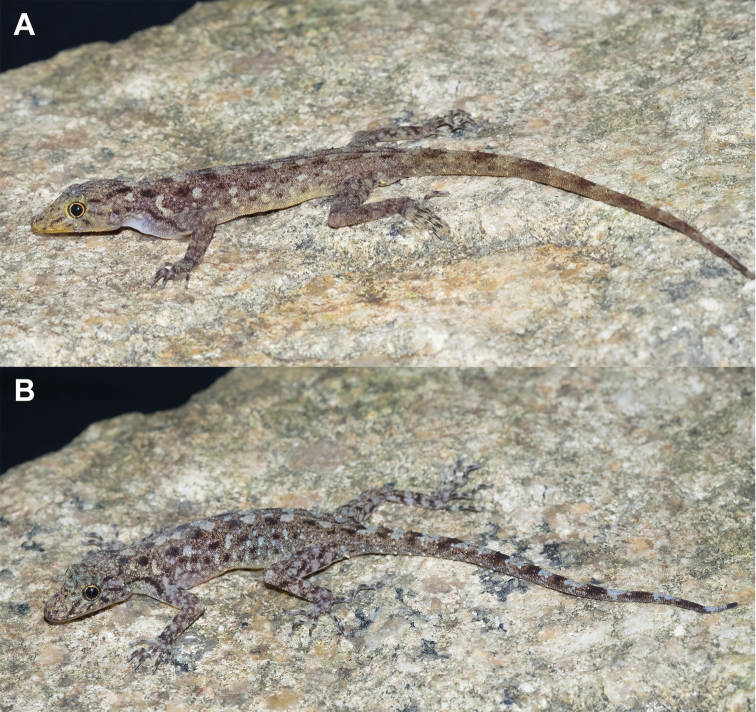
*Cnemaspisadangrawi* sp. nov. from Adang Island, Mueang Satun District, Satun Province, Thailand **A** male holotype ZMKU R 00767 **B** female paratype ZMKU R 00768.

**Figure 9.**
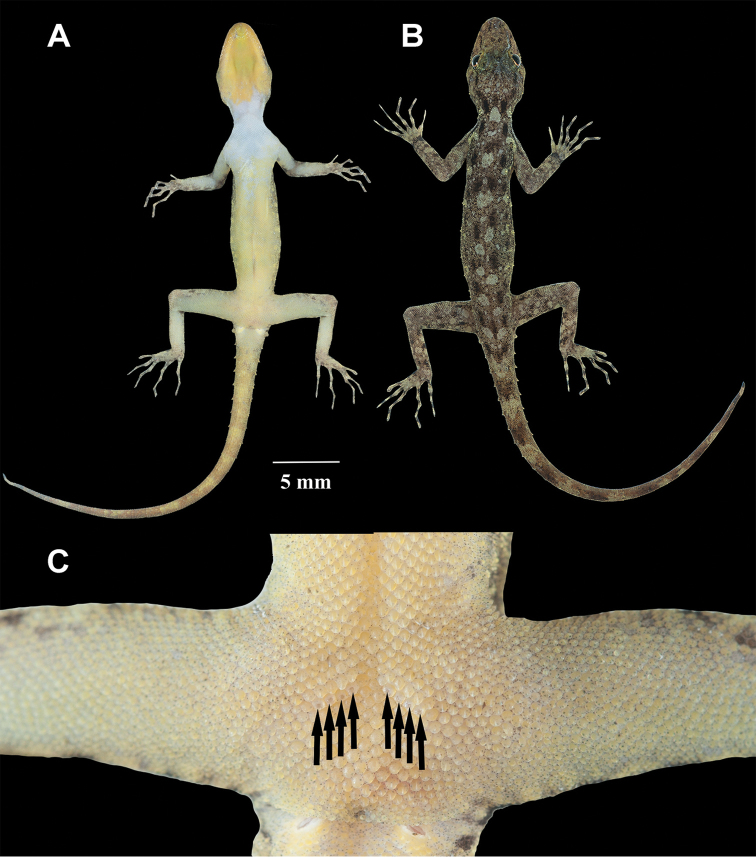
Male holotype of *Cnemaspisadangrawi* sp. nov. from Adang Island, Mueang Satun District, Satun Province, Thailand (ZMKU R 00767) in life. **A** ventral view **B** dorsal view **C** precloacal region showing distribution of pore-bearing scales (black arrows).

**Figure 10.**
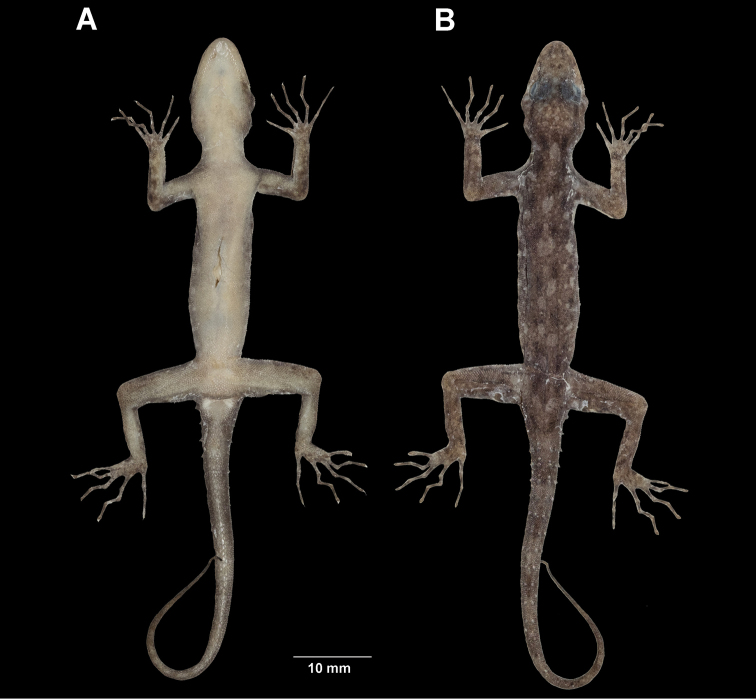
Holotype (adult male) of *Cnemaspisadangrawi* sp. nov. from Adang Island, Mueang Satun District, Satun Province, Thailand (ZMKU R 00767) in preservative. **A** ventral **B** dorsal views.

(Figs 8a, 9, 10). ZMKU R 00767, adult male from Thailand, Satun Province, Mueang Satun District, Tarutao National Park, Adang Island, Jonsalad Waterfall (6°30.7806'N, 99°18.0072'E; 84 m a.s.l.; Fig. 13A), collected on 9 November 2017 by Natee Ampai, Attapol Rujirawan, Siriporn Yodthong, and Korkwan Termprayoon.

##### Paratypes

**Figure 11.**
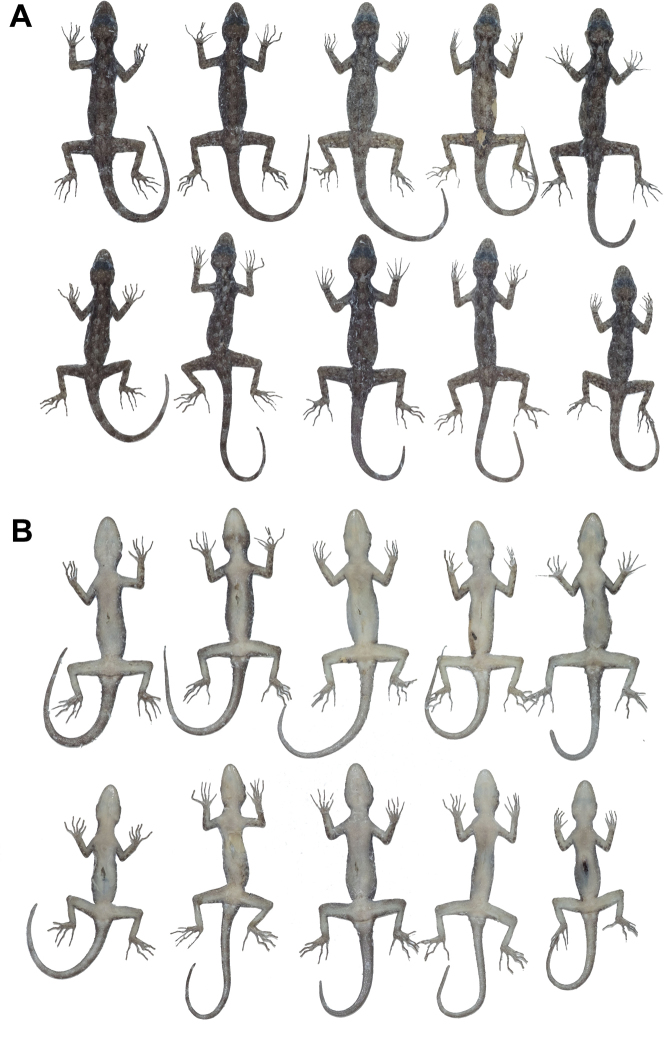
Male paratypes of *Cnemaspisadangrawi* sp. nov. in preservative. **A** dorsal view **B** ventral view; from left to right, top panel: ZMKU R 00769, ZMKU R 00770, ZMKU R 00773, ZMKU R 00775, and ZMKU R 00776; bottom panel: THNHM 28206, THNHM 28207, THNHM 28208, THNHM 28209, and THNHM 28210.

**Figure 12.**
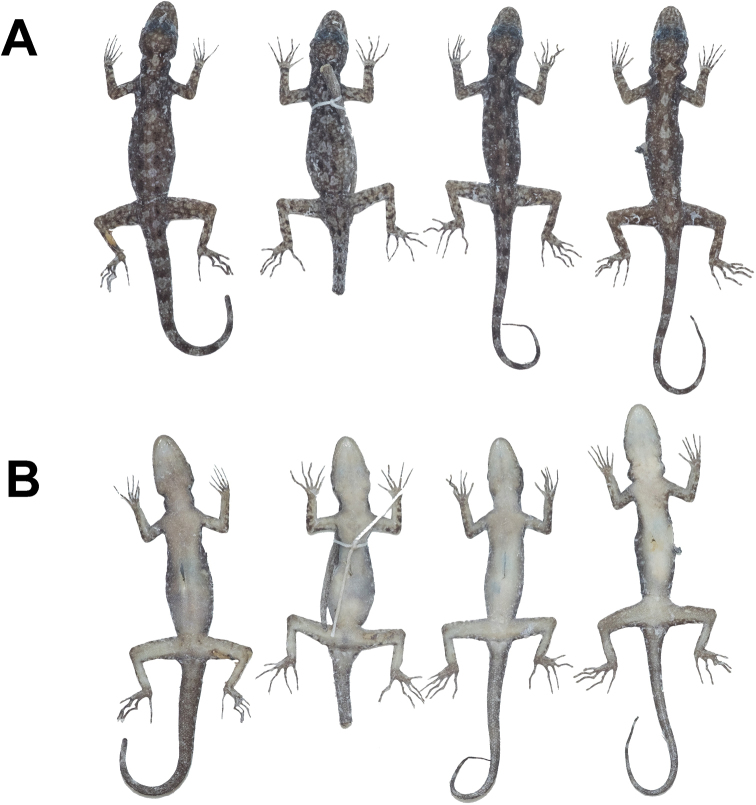
Female paratypes of *Cnemaspisadangrawi* sp. nov. in preservative. **A** dorsal view **B** ventral view; from left to right: ZMKU R 00774, THNHM 28211, ZMKU R 00768, and ZMKU R 00771.

**Figure 13.**
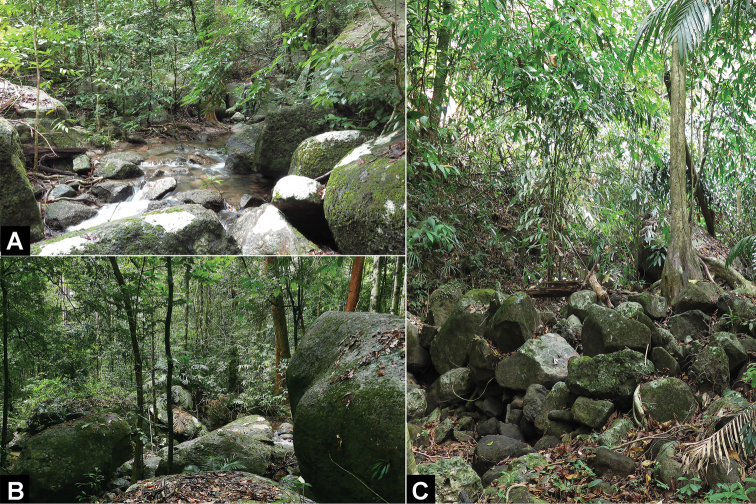
Habitats of *Cnemaspisadangrawi* sp. nov. **A** Jonsalad Waterfall at type locality of Adang Island **B** habitat of paratypes in outcropped near stream at Rawi Island **C** habitat of paratypes in forest stream near mangrove at Rawi Island, Mueang Satun district, Satun Province, Thailand.

(Figs 8b, 11, 12). Fourteen paratypes (adult males = 10, adult females = 4). ZMKU R 00768 (1 adult female), same locality and collectors as holotype. ZMKU R 00771 (1 adult female), and ZMKU R 00769–00770, THNHM 28206–28209 (6 adult males), same data as holotype except collected 6 April 2018. ZMKU R 00773, ZMKU R 00775, THNHM 28210 (3 adult males) and ZMKU R 00774 (1 adult female), same collectors as holotype except from Rawi Island (6°33.9084'N, 99°15.5088'E; 7 m a.s.l.; Fig. 13B), collected on 7 April 2018. ZMKU R 00776 (1 adult male) and THNHM 28211 (1 adult female), same collectors as holotype except from Rawi Island (6°33.3474'N, 99°15.0018'E; 7 m a.s.l.; Fig. 13C), collected on 8 April 2018.

##### Referred specimens.

ZMKU R 00772 and THNHM 28212–28215 (five juveniles), same data as holotype except collected 6 April 2018.

##### Diagnosis.

*Cnemaspisadangrawi* sp. nov. can be distinguished from all other *Cnemaspis* by having the following combination of characters: (1) adult males with maximum SVL length 44.9 mm (mean 41.8 ± SD 2.5, *n* = 11) and females with maximum SVL 43.8 mm (mean 42.5 ± SD 1.5, *n* = 4); (2) 10 supralabials and 9 infralabials; (3) 6–8 pore-bearing precloacal scales with rounded pores arranged in chevron shape and separated; (4) 23–25 paravertebral tubercles randomly arranged; (5) 26–28 subdigital lamellae under the 4^th^ toe; (6) subcaudal scales keeled and lacking enlarge median row; (7) one postcloacal tubercle each side; (8) gular region, abdomen, limbs and subcaudal region yellowish in males only; (9) mid-gular marking absent in males and females. These differences are summarized for geographically close congeners in the *siamensis* group (Table 6).

**Table 6.** Meristic character state and color pattern of species in the *Cnemapsissiamensis* group. Measurements are taken in millimeters and measurement abbreviations are defined in the text. – = data unavailable, w = weak.

Characters/Species	*C.adangrawi* sp. nov.	*C.chanardi*	*C.huaseesom*	*C.omari*	*C.phangngaensis*	*C.punctatonuchalis*	*C.roticanai*	*C.siamensis*	*C.thachanaensis*	*C.vandeventeri*
Sample size	15	25	5	8	2	5	8	12	6	3
Maximum SVL	44.9	40.9	43.5	41.3	42.0	49.6	47.0	39.7	39.0	44.7
Supralabial scales	10	8–10	7–10	8–9	10	8	8–9	8–9	10–11	8,9
Infralabial scales	9	8	6–9	7–8	10	7–8	7–8	6–8	9–11	7–9
Ventral scales keeled (1) or smooth (0)	1	1	0	1	1	0	1	1	1	1
No. of precloacal pores	6–8	6–8	5–8	3–6	4	0	3–6	0	0	4
Precloacal pore continuous (1) or separated (0)	0	0	1	0	1	–	0	–	–	0
Precloacal pores elongate (1) or round (0)	0	0	0	0	0	–	0	–	–	0
No. of paravertebral tubercles	23–25	22–25	18–24	22–29	22	24–27	25–27	19–25	15–19	25–29
Paravertebral tubercles linearly arranged (1) or more random (0)	0	0	w,0	w,0	1	w	0	0	1	0
Tubercles present (1) or absent (0) on lower flanks	0	1	1	w,1	0	1	1	1	1	1
No. of 4^th^ toe lamellae	26–28	26–29	21–31	25–28	29	29–31	26–29	24–26	24	24–28
Lateral caudal furrows present (1) or absent (0)	1	1	1	1	1	1	1	1	1	0
Subcaudal keeled (1) or smooth (0)	1	1	0	1	1	0	1	1	1	1
Enlarge median subcaudal scales row (1) or not (0)	0	1	0	0	0	1	w	1	0	1
No. of postcloacal tubercles in males	1	1	1,2	1	2	1–3	1–2	1–2	0	1–3
Subtibial scales keeled (1) or smooth (0)	1	1	0	1	1	1	1	1	1	1
Subcaudal region yellow present (1) or not (0)	1	1	1	1	1	0	1	0	0	0
Ventral pattern sexually dimorphic present (1) or not (0)	1	1	1	–	0	1	1	1	1	1
Dorsal color pattern sexually dimorphic (1) or not (0)	0	0	1	0	0	1	1	0	0	0
Lineate gular marking (1) or not (0)	0	0	0	0	0	–	0	1	1	0

##### Description of holotype.

Adult male; SVL 44.6 mm; head moderately sized (HL/SVL 0.26), narrow (HW/SVL 0.15), flattened (HD/HL 0.38), and head distinct from neck; snout moderate (ES/HL 0.47), snout slightly concave in lateral view; postnasal region constricted medially; scales of rostrum smooth, larger than conical scales on occiput; weak supraorbital ridges; lineate gular marking absent; gular and throat scales raised, keeled and round; shallow frontorostral sulcus; canthus rostralis nearly absent, smoothly rounded; eye large (ED/HL 0.20); pupil round; ear opening oval, taller than wide; rostral slightly concave; rostral bordered posteriorly by supranasals; 10, 10 (right, left) supralabials decreasing in size posteriorly; 9, 9 (right, left) infralabials decreasing in size posteriorly; nostril elliptical, oriented posterodorsally, bordered by small postnasal scales; mental large, triangular, concave bordered posteriorly by three large postmentals.

Body slender, elongate (AG/SVL 0.42); small, keeled, dorsal scales equal in size throughout body intermixed with several large, keeled, multicarinate tubercles randomly arranged; 24 paravertebral tubercles; tubercles absent on lower flanks; tubercles extend from occiput to base of tail; dorsal scales raised and keeled; pectoral and abdominal scales keeled, round, flat to concave, slightly larger than dorsal and not larger posteriorly; ventral scales of brachia smooth, raised and juxtaposed; eight separated pore-bearing precloacal scales with rounded pores; precloacal depression absent; femoral pores absent.

Fore and hind limbs moderately long, slender; scales beneath forearm slightly raised, smooth and subimbricate; subtibial scales keeled; palmar scales smooth and juxtaposed; digits elongate, slender, inflected joint and bearing slightly recurved claws; subdigital lamellae unnotched; lamellae beneath first phalanges wide; lamellae beneath phalanx immediately following inflection granular; lamellae of distal phalanges wide; lamellae beneath inflection large; interdigital webbing absent; enlarged submetatarsal scales on 1^st^ toe absent; fingers increase in length from first to fourth with fourth and fifth nearly equal in length; relative length of fingers IV>V>III>II>I; toes increase in length from first to fifth with fourth and fifth nearly equal in length; relative length of toes IV>V>III>II>I; total subdigital lamellae on 4^th^ toe 28, 28 (right, left).

Caudal and subcaudal scales keeled, similar to dorsal scale size; lateral caudal furrow present; enlarge caudal tubercles arranged in segmented whorls, not encircling tail; enlarge median subcaudal scales row absent; caudal tubercles present on lateral furrow; tail length (TL) 58.3 mm with regenerated tail; enlarge, flat, postcloacal tubercle 1, 1 (right, left) on lateral surface of hemipenial swellings at the base of tail.

##### Coloration in life

(Figs 8, 9). Dorsal ground color of head light brown, top of head bearing small, faint black and yellowish markings; thin, black postorbital stripes extending to nape; light-colored prescapular cresent; dorsal ground color of body, limbs and tail light brown with black irregular blotches; ground color of ventral surfaces grayish-white intermixed with yellowish blotches; ventral pattern sexually dimorphic, anterior gular region, abdominal region, and caudal region yellowish in males; two dark blotches on nape form a bipartite pattern; light sage vertebral blotches extending from the nape to tail; flanks with irregular incomplete brown to yellowish blotches becoming smaller posteriorly; tubercles on anterior and posterior of the body were white or yellow; widely separated, white or yellow tubercles occur on flanks; limbs beige with dark brown mottling; tail faintly marked with dark brown.

##### Coloration in preservative

(Fig. 10). Color pattern similar to that in life with some fading of markings. Dorsal ground color of head, body, limbs and tail brown, darker with indistinct, irregular markings. All yellow coloration in gular region, ventral surfaces, flanks and tail faded to creamy white.

##### Variation.

Most paratypes approximate the holotype in general aspects of morphology (Figs 11, 12), with most differences found in the degree of vertebral blotches. All adult female paratypes lack yellowish coloration in the gular, abdominal, and caudal regions. ZMKU R 00767, THNHM 28208, THNHM 28210, and ZMKU R 00776 (four adult males) have regenerated tails of uniform tan coloration. THNHM 28207–28209, ZMKU R 00773, and ZMKU R 00775 (five adult males) have lighter dorsal markings that appear more as transverse bands than as paravertebral blotches. THNHM 28211 (one adult female) has a broken tail. Differences in meristic and morphometrics within the type series are presented in Table 7.

**Table 7.** Descriptive measurements in millimeters and characters of the type series of *Cnemaspisadangrawi* sp. nov. M = male; F = female; – = data unavailable or absent; b = broken; r = regenerated.

**Museum number**	**ZMKU**	**ZMKU**	**THNHM**	**THNHM**	**THNHM**	**ZMKU**	**THNHM**	**ZMKU**	**ZMKU**	**THNHM**	**ZMKU**	**ZMKU**	**ZMKU**	**ZMKU**	**THNHM**
	**R 00767**	**R 00769**	**28206**	**28207**	**28208**	**R 00770**	**28209**	**R 00773**	**R 00775**	**28210**	**R 00776**	**R 00768**	**R 00771**	**R 0074**	**28211**
Type series	Holotype	Paratype	Paratype	Paratype	Paratype	Paratype	Paratype	Paratype	Paratype	Paratype	Paratype	Paratype	Paratype	Paratype	Paratype
Sex	M	M	M	M	M	M	M	M	M	M	M	F	F	F	F
SVL	44.6	44.9	37.9	39.7	43.7	42.2	41.5	42.8	42.3	37.6	43.1	40.6	43.8	43.7	41.9
TL	58.3r	55.7	47.7	53.1	39.7r	56.5	49.8	56.2	51.0	45.6r	42.3r	50.5	50.3	42.6	40.5b
TW	4.3	4.3	3.9	3.8	4.3	4.1	4.0	4.1	4.0	3.8	4.2	4.0	4.1	4.3	4.1
FL	6.6	6.6	5.9	6.0	6.6	6.4	6.3	6.4	6.4	5.9	6.5	6.2	6.5	6.5	6.4
TBL	8.5	8.5	7.7	8.1	8.5	8.4	8.3	8.3	8.4	7.6	8.4	8.1	8.4	8.4	8.3
AG	18.8	18.7	15.2	17.2	18.5	18.3	18.3	18.4	18.3	15.2	18.5	18.3	18.6	18.6	18.4
HL	11.6	11.6	10.9	10.9	11.6	11.2	11.0	11.2	11.2	10.6	11.4	11.0	11.1	11.3	11.1
HW	7.0	7.0	6.4	6.5	7.0	6.8	6.7	6.8	6.8	6.4	6.9	6.6	6.7	6.9	6.8
HD	4.5	4.2	3.9	3.9	4.5	4.3	4.0	4.5	4.5	2.8	4.5	4.2	4.4	4.5	4.4
ED	2.4	2.4	2.1	1.9	2.4	2.2	2.1	2.3	2.3	2.0	2.4	2.2	2.4	2.4	2.2
EE	3.7	3.8	3.2	3.3	3.6	3.8	3.6	3.8	3.7	3.3	3.7	3.5	3.8	3.8	3.7
ES	5.5	5.4	4.5	4.6	5.2	5.2	5.0	5.1	5.1	4.4	5.1	5.0	5.2	5.2	5.0
EN	4.4	4.3	3.3	3.4	4.1	4.0	4.0	4.2	4.1	3.2	4.2	4.2	4.1	4.2	4.1
IO	2.9	2.9	2.6	2.6	2.9	2.8	2.7	2.8	2.8	2.7	2.8	2.7	2.9	2.8	2.7
EL	0.9	0.9	0.8	0.8	0.9	0.9	0.9	0.8	0.9	0.8	0.9	0.8	0.9	0.9	0.8
IN	0.9	0.9	0.9	0.9	0.9	0.9	0.9	0.9	0.8	0.8	0.9	0.8	0.8	0.9	0.9
Supralabials	10	10	10	10	10	10	10	10	10	10	10	10	10	10	10
Infralabials	9	9	9	9	9	9	9	9	9	9	9	9	9	9	9
No. of precloacal pores	8	6	6	6	6	6	6	8	6	8	6	–	–	–	–
Precloacal pore continuous (1) or separated (0)	0	0	0	0	0	0	0	0	0	0	0	–	–	–	–
No. of paravertebral tubercles	24	25	25	24	25	24	25	23	24	23	25	25	25	23	23
No. of 4th toe lamellae	28	28	27	28	28	27	28	27	27	27	28	26	28	26	28

##### Distribution and natural history.

*Cnemaspisadangrawi* sp. nov. is known only from Adang and Rawi islands, 60 and 61 km off the coast of Thailand, respectively (Fig. 1). All Adang specimens were found in a granitic rocky stream (Fig. 13A). Rawi Island specimens were found in rock outcrops along a stream (Fig. 13B) and along a forest stream near mangroves (Fig. 13C). Sixteen specimens (ZMKU R 00767–00768, ZMKU R 00770–00772, ZMKU R 00775–00776, THNHM 28206–28209, and THNHM 28211–28215) were collected during the day (1047–1823 h) and four specimens (ZMKU R 00769, ZMKU R 00773–00774, and THNHM 28210) were collected at night (1927–2024 h). The male holotype was found during the day (1047 h) on the base of a rock boulder with holes formed by the expansive soil between the ground and rock interface of a nearby stream.

Paratypes found during the day (ZMKU R 00767–00768, ZMKU R 00770–00772, ZMKU R 00775–00776, THNHM 28206–28209, and THNHM 28211–28215) were in crevices of boulders, shaded areas with holes in the soil at the base of a rock wall near a stream, and on boulder outcrops near streams. When disturbed, some individuals would retreat into rock crevices or into holes in the soil at the base of a rock wall. Paratypes found at night (ZMKU R 00769, ZMKU R 00773–00774 and THNHM 28210) were in shaded areas (by day), deep at the base of boulders, or perched on vegetation near a rocky stream. Two gravid females (ZMKU R 00771 and THNHM 28211) contained one or two eggs during November 2017. Some juveniles (not collected) were found in holes in the soil and perched on vegetation near a stream at Rawi Island on 8 April 2018. At night, *Cyrtodactylusmacrotuberculatus* was found in syntopy on the rock wall and vegetation near a stream at Jonsalad Waterfall, Adang Island, with *Cnemaspisadangrawi* sp. nov.

##### Etymology.

The specific epithet refers to Adang and Rawi islands where the new species is found, and is a noun in apposition.

##### Comparisons.

*Cnemaspisadangrawi* sp. nov. can be distinguished from other members of the *siamensis* group (*C.chanardi*, *C.huaseesom*, *C.omari*, *C.phangngaensis*, *C.punctatonuchalis*, *C.roticanai*, *C.siamensis*, *C.thachanaensis*, and *C.vandeventeri*) by having a smaller maximum SVL of 44.9 mm (vs 47.0 mm in *C.roticanai*, 49.6 mm in *C.punctatonuchalis*) and by having a larger maximum SVL 44.9 mm (vs 40.9 mm in *C.chanardi*, 43.5 mm in *C.huaseesom*, 41.3 mm in *C.omari*, 42.0 mm in *C.phangngaensis*, 39.7 mm in *C.siamensis*, 39.0 mm in *C.thachanaensis*, and 44.7 mm in *C.vandeventeri*).

*Cnemaspisadangrawi* sp. nov. is distinguished from *C.omari*, *C.punctatonuchalis*, *C.roticanai*, *C.siamensis*, and *C.vandeventeri* by having 10 supralabial scales (vs eight in *C.punctatonuchalis* and 8–9 in *C.omari*, *C.roticanai*, *C.siamensis*, and *C.vandeventeri*). This species is distinguished from *C.chanardi*, *C.omari*, *C.phangngaensis*, *C.punctatonuchalis*, *C.roticanai*, and *C.siamensis* by having 9 infralabial scales (vs 8 in *C.chanardi*, 7–8 in *C.omari*, *C.punctatonuchalis*, *C.roticanai*, 10 in *C.phangngaensis*, and 6–8 in *C.siamensis*). This species is distinguished from *C.huaseesom* and *C.punctatonuchalis* by having keeled ventral and subcaudal scales (vs smooth ventral and subcaudal scales in *C.huaseesom* and *C.punctatonuchalis*).

*Cnemaspisadangrawi* sp. nov. is distinguished from *C.phangngaensis* and *C.vandeventeri* by having 6–8 precloacal pores (vs 4 in *C.phangngaensis* and *C.vandeventeri*). This species is distinguished from *C.punctatonuchalis*, *C.siamensis*, and *C.thachanaensis* by presence of precloacal pores (vs precloacal pores absent in *C.punctatonuchalis*, *C.siamensis*, and *C.thachanaensis*). This species is distinguished from *C.huaseesom* and *C.phangngaensis* by having a separated row of precloacal pores (vs continuous in *C.huaseesom* and *C.phangngaensis*).

*Cnemaspisadangrawi* sp. nov. is distinguished from *C.phangngaensis* and *C.thachanaensis* by having 23–25 paravertebral tubercles (vs 22 in *C.phangngaensis* and 15–19 in *C.thachanaensis*). This species is distinguished from *C.huaseesom*, *C.omari*, *C.punctatonuchalis*, *C.roticanai*, *C.siamensis*, *C.thachanaensis*, and *C.vandeventeri* by lacking tubercles on lower flanks (vs present in *C.huaseesom*, *C.omari*, *C.punctatonuchalis*, *C.roticanai*, *C.siamensis*, *C.thachanaensis*, and *C.vandeventeri*). This species is distinguished from *C.phangngaensis*, *C.punctatonuchalis*, and C. *thachanaensis* by having 26–28 lamellae under 4^th^ toe (vs 29 in *C.phangngaensis*, 29–31 in *C.punctatonuchalis*, and 24 in *C.thachanaensis*). This species is distinguished from *C.chanardi* and *C.vandeventeri* by having lateral caudal furrows (vs lacking in *C.chanardi* and *C.vandeventeri*).

*Cnemaspisadangrawi* sp. nov. can be further distinguished from *C.chanardi*, *C.punctatonuchalis*, *C.siamensis*, and *C.vandeventeri* by lacking enlarged median subcaudal scales (vs present in *C.chanardi*, *C.punctatonuchalis*, *C.siamensis*, and *C.vandeventeri*). This species is distinguished from *C.huaseesom* by having keeled subtibial scales (vs smooth subtibial scales in *C.huaseesom*). This species is distinguished from *C.siamensis* and *C.thachanaensis* by lacking lineate gular marking (vs present in *C.siamensis* and *C.thachanaensis*).

## Discussion

Studies on the taxonomy and systematics of *Cnemaspis* in Southeast Asia have increased in the past two decades (Bauer and Das 1998; Das 2005; Bauer et al. 2007; Grismer and Chan 2010; Wood et al. 2013; Grismer et al. 2014; Iskandar et al. 2017; Wood et al. 2017). Integrative taxonomic approaches that incorporated both morphological and molecular data have been especially useful in uncovering cryptic diversity of Thai *Cnemaspis* (Grismer et al. 2014; Wood et al. 2017). Our descriptions of *C.tarutaoensis* sp. nov. and *C.adangrawi* sp. nov. bring the total number of *Cnemaspis* to 59 species, of which 18 occur in Thailand.

Previously, the reported geographic distribution of *Cnemaspis* in Thailand was mostly restricted to the mainland (Smith 1925; Taylor 1963; Bauer and Das 1998; Grismer et al. 2010; Wood et al. 2017), with insular populations of *Cnemaspis* known only from four localities in Thailand, including Samui, Phangan and Ko Tao islands, Surat Thani Province (Gulf of Thailand; approximately 85 km offshore the mainland of Mueang Chumphon District, Chumphon Province; Grismer et al. 2010, 2014) and Phuket Island, Phuket Province (Andaman Sea; approximately 30 km offshore the mainland of Takua Thung District, Phangnga Province; Das and Leong 2004). The descriptions of *C.tarutaoensis* sp. nov. and *C.adangrawi* sp. nov. double the number of *Cnemaspis* species known from islands in Thailand from two (*C.chanardi* and *C.siamensis*) to four. These two new species occur on Tarutao, Adang and Rawi islands in the Andaman Sea, offshore of the Thai mainland of Mueang Satun District, Satun Province (at approximately 40 km, 60 km, and 61 km, respectively). These islands were connected to the mainland during the last glacial maximum as recently as 21,000 years before present (Voris 2000; Sathiamurthy and Voris 2006), but the timing of their divergence from other *Cnemaspis* species remains untested.

The complex geological history in Thailand created a large number of limestone and granitic formations in southern Thailand (Day and Urich 2000; Morley et al. 2011). The karst regions and granitic rocky streams of southern Thailand are proving to harbor a high diversity of range-restricted species of geckos (Smith 1925; Taylor 1963; Grismer et al. 2010, 2014; Wood et al. 2017). Further research and additional field surveys in unexplored karst regions on islands and the mainland are needed to better understand the taxonomy, ecology, distribution, biogeography, and conservation of *Cnemaspis* in southern Thailand.

## Supplementary Material

XML Treatment for Cnemaspis tarutaoensis

XML Treatment for Cnemaspis adangrawi

## Appendix 1

List of comparative specimens examined.

*Cnemaspischanardi*: Thailand, Trang Province, Nayong District, Ban Chong: THNHM 06983 (male holotype); Krabi Province, Klong Thom District: THNHM 012439–012440 (males); Mueang Krabi District: THNHM 012436 and 012437 (males), THNHM 012438 (female); Nakhon Si Thammarat Province, Tha Sala District: THNHM 020992 (male); Lansaka district: THNHM 014111 (immature male); Noppitam district: THNHM 013838 (male), THNHM 010705 (male); Surat Thani Province, Ang Thong Island, Mueang Surat Thani District: THNHM 016074 (female).

*Cnemaspishuaseesom*: Thailand, Kanchanaburi Province, Sai Yok District, Sai Yok National Park: THNHM 15909 (male holotype).

*Cnemaspisniyomwanae*: Thailand, Trang Province, Palean District, Thum Khao Ting: THNHM 15909 (female holotype).

*Cnemaspispunctatonuchalis*: Thailand, Prachuap Khiri Khan Province, Thap Sakae District, Huay Yang National Park: THNHM 02001 (male holotype)

*Cnemaspissiamensis*: Thailand, Nakhon Si Thammarat Province, Lansaka District: THNHM 013828 (male); Tha Sala District: THNHM 018265 (male); Phetchabun Province, Nam Nao District: THNHM 01336 (female), THNHM 01337 (male); Phetchaburi Province, Cha-am District: THNHM 01448 (male), THNHM 01449 (immature male); Chumpon Province, Mueang Chumpon District: THNHM 0372 (male); Phato District: THNHM 01086 (male); Surat Thani Province, Vibhawadee District: THNHM 01084 (female); Ang Thong Island, Mueang Surat Thani District: THNHM 015624 (female).

*Cnemaspisvandeventeri*: Thailand, Ranong Province, Kapur District, Klong Naka: THNHM 08261 (male holotype), THNHM 08260 (female).

## References

[B1] Bauer AM Das I (1998) A new *Cnemaspis* (Reptilia: Gekkonidae) from Southeastern Thailand. Copeia 1998: 439–444. 10.2307/1447438

[B2] Bauer AM De Silva A Greenbaum E Jackman T (2007) A new species of day gecko from high elevation in Sri Lanka, with a preliminary phylogeny of Sri Lankan *Cnemaspis* (Reptilia: Squamata: Gekkonidae). Mitteilungen aus dem Museum für Naturkunde, Berlin, Zoologische Reihe 83: 22–32. 10.1002/mmnz.200600022

[B3] Bauer AM Giri VB Greenbaum E Jackman T Dharne MS Shouche SY (2008) On the systematics of the gekkonid genus *Teratolepis* Günther, 1869: another one bites the dust. Hamadryad 32(2): 90–104.

[B4] Das I (2005) Revision of the genus *Cnemaspis* Strauch, 1887 (Sauria: Gekkonidae), from the Mentawai and Adjacent Archipelagos of Western Sumatra, Indonesia, with description of four new species. Journal of Herpetology 39(2): 233–247. 10.1670/61-02A

[B5] Das I Leong TM (2004) A new species of *Cnemaspis* (Sauria: Gekkonidae) from Southern Thailand. Current Herpetology 23(2): 63–71. 10.5358/hsj.23.63

[B6] Das I van Dijk PP (2013) Species richness and endemicity of the herpetofauna of South and Southeast Asia. Raffles Bulletin of Zoology 29: 269–277.

[B7] Day M Urich P (2000) An assessment of protected karst landscapes in Southeast Asia. Cave and Karst Science 27: 61–70.

[B8] Grismer LL Chan KO (2010) Another new rock gecko (genus *Cnemaspis* Stauch 1887) from Pulau Langkawi, Kedah, Peninsular Malaysia. Zootaxa 2419: 51–62. 10.11646/zootaxa.2419.1.2

[B9] Grismer LL Norhayati A Chan KO Daicus B Muin MA Wood Jr PL Grismer JL (2009) Two new diminutive species of *Cnemaspis* Strauch 1887 (Squamata: Gekkonidae) from Peninsular Malaysia. Zootaxa 2019: 40–56. 10.5281/zenodo.186045

[B10] Grismer LL Sumontha M Cota M Grismer JL Wood Jr PL Pauwels OS Kunya K (2010) A revision and redescription of the rock gecko *Cnemaspissiamensis* (Taylor 1925) (Squamata: Gekkonidae) from Peninsular Thailand with descriptions of seven new species. Zootaxa 2576: 1–55. 10.11646/zootaxa.2576.1.1

[B11] Grismer LL Wood Jr PL Tri N Murdoch ML (2015a) The systematics and independent evolution of cave ecomorphology in distantly related clades of Bent-toed Geckos (Genus *Cyrtodactylus* Gray, 1827) from the Mekong Delta and islands in the Gulf of Thailand. Zootaxa 3980(1): 106–126. 10.11646/zootaxa.3980.1.6 26249941

[B12] Grismer LL Wood Jr PL Quah ES Anuar S Ngadi E Norhayati A (2015b) A new insular species of Rock Gecko (*Cnemaspis* Boulenger) from Pulau Langkawi, Kedah, Peninsular Malaysia. Zootaxa 3985(2): 203–218. 10.11646/zootaxa.3985.2.2 26250030

[B13] Grismer LL, Wood Jr PL, Shahrul A, Awal R, Norhayati A, Muin M, Sumontha M,

[B14] Grismer J Chan K Quah ES Pauwels O (2014) Systematics and natural history of Southeast Asian Rock Geckos (genus *Cnemaspis* Strauch, 1887) with descriptions of eight new species from Malaysia, Thailand, and Indonesia. Zootaxa 3880(1): 1–147. 10.11646/zootaxa.3880.1.1 25544645

[B15] Huelsenbeck JP Ronquist F (2001) MRBAYES: Bayesian inference of phylogeny. Bioinformatics 17: 754–755. 10.1093/bioinformatics/17.8.754 11524383

[B16] Hughes JB Round PD Woodruff DS (2003) The Indochinese-Sundaic faunal transition at the Isthmus of Kra: an analysis of resident forest bird species distributions. Journal of Biogeography 30(4): 569–580. 10.1046/j.1365-2699.2003.00847.x

[B17] Iskandar DT McGuire JA Amarasinghe AAT (2017) Description of five new day geckos of *Cnemaspiskandiana* Group (Sauria: Gekkonidae) from Sumatra and Mentawai Archipelago, Indonesia. Journal of Herpetology 51(1): 142–153. 10.1670/15-047

[B18] Kalyaanamoorthy S Minh BQ Wong TK von Haeseler A Jermiin LS (2017) ModelFinder: fast model selection for accurate phylogenetic estimates. Nature Methods 14: 587–589. 10.1038/nmeth.4285 28481363 PMC5453245

[B19] Kumar SSG Stecher G Tamura K (2016) MEGA7: molecular evolutionary genetics analysis version 7.0 for bigger data sets. Molecular Biology and Evolution 33(7): 1870–1874. 10.1093/molbev/msw054 27004904 PMC8210823

[B20] Macey JR Larson A Ananjeva NB Fang Z Papenfuss TJ (1997) Two novel gene orders and the role of light-strand replication in rearrangement of the vertebrate mitochondrial genome. Molecular Biology and Evolution 14: 91–104. 10.1093/oxfordjournals.molbev.a025706 9000757

[B21] Miller MA Pfeiffer W Schwartz T (2010) Creating the CIPRES Science Gateway for inference of large phylogenetic trees. Proceedings of the Gateway Computing Environments Workshop (GCE), 14 Nov. 2010, New Orleans, LA, 1–8. 10.1109/GCE.2010.5676129 [Accessed on: 2018-11-27]

[B22] Minh Q Nguyen M von Haeseler AA (2013) Ultrafast approximation for phylogenetic bootstrap. Molecular Biology and Evolution 30: 1188–1195. 10.1093/molbev/mst024 23418397 PMC3670741

[B23] Morley CK Charusiri P Watkinson I (2011) Structural geology of Thailand during the Cenozoic. In: Ridd MF Barber AJ Crow MJ (Eds) The Geology of Thailand.The Geological Society, London, 273–334. 10.1144/GOTH.11

[B24] Myers N Mittermeier RA Mittermeier CG Fonseca GAB Kent J (2000) Biodiversity hotspots for conservation priorities. Nature 403: 853–858. 10.1038/35002501 10706275

[B25] Nguyen LT Schmidt HA von Haeseler A Minh BQ (2014) IQ-TREE: a fast and effective stochastic algorithm for estimating maximum-likelihood phylogenies. Molecular Biology and Evolution 32: 268–274. 10.1093/molbev/msu300 25371430 PMC4271533

[B26] Parnell J (2013) The biogeography of the Isthmus of Kra region: a review. Nordic Journal of Botany 31(1): 1–15. 10.1111/j.1756-1051.2012.00121.x

[B27] Posada D (2008) jModelTest: Phylogenetic model averaging. Molecular Biology and Evolution 25(7): 1253–1256. 10.1093/molbev/msn083 18397919

[B28] Rambaut A (2009) FigTree version 1.4.3. http://tree.bio.ed.ac.uk/software/figtree.

[B29] Rambaut A Suchard MA Xie D Drummond AJ (2014) Tracer v1.6. http://beast.bio.ed.ac.uk/Tracer [Accessed on: 2018-8-25]

[B30] Riyanto A Hamidy A Sidik I Gunalen D (2017) A new species of rock gecko of the genus *Cnemaspis* Strauch, 1887 (Squamata: Gekkonidae) from Belitung Island, Indonesia. Zootaxa 4358: 583–597. 10.11646/zootaxa.4358.3.12 29245465

[B31] Sathiamurthy E Voris HK (2006) Maps of Holocene sea level transgression and submerged lakes on the Sunda Shelf. The Natural History Journal of Chulalongkorn University 2 (Supplement): 1–43.

[B32] Siler CD Oaks JR Esselstyn JA Diesmos AC Brown RM (2010) Phylogeny and biogeography of Philippine bent-toed geckos (Gekkonidae: *Cyrtodactylus*) contradict a prevailing model of Pleistocene diversification. Molecular Phylogenetics and Evolution 55(2): 699–710. 10.1016/j.ympev.2010.01.027 20132898

[B33] Smith MA (1925) Contribution to the herpetology of Borneo. The Sarawak Museum Journal 3(8): 15–34.

[B34] Sodhi NS Koh LP Brook BW Ng PKL (2004) Southeast Asian biodiversity: an impending disaster. Trends in Ecology and Evolution 19: 654–660. 10.1016/j.tree.2004.09.006 16701328

[B35] Strauch AA (1887) Bemerkungen über die Geckoniden-Sammlung im zoologischen Museum der kaiserlichen Akademie der Wissenschaften zu St. Petersburg. Mémoires des Savants Étrangers 7: 1–72.

[B36] Taylor EH (1963) The lizards of Thailand. The University of Kansas Science Bulletin 44: 687–1077.

[B37] Trifinopoulos J Nguyen LT von Haeseler A Minh BQ (2016) W-IQ-TREE: a fast online phylogenetic tool for maximum likelihood analysis. Nucleic Acids Research 44 (W1): W232–W235. 10.1093/nar/gkw256 PMC4987875 27084950

[B38] Uetz P Freed P Hošek J (2018) The Reptile Database. http://www.reptile-database.org. [Accessed on: 2019-1-29]

[B39] Voris HK (2000) Maps of pleistocene sea levels in Southeast Asia: shorelines, river systems and time durations. Journal of Biogeography 27(5): 1153–1167. 10.1046/j.1365-2699.2000.00489.x

[B40] Wilcox TP Zwickl DJ Heath TA Hillis DM (2002) Phylogenetic relationships of the dwarf boas and a comparison of Bayesian and bootstrap measures of phylogenetic support. Molecular Phylogenetics and Evolution 25: 361–371. 10.1016/S1055-7903(02)00244-0 12414316

[B41] Wood Jr PL Grismer LL Aowphol A Aguilar CA Cota M Grismer MS Murdoch ML Sites Jr JW (2017) Three new karst-dwelling *Cnemaspis* Strauch, 1887 (Squamata; Gekkonidae) from Peninsular Thailand and the phylogenetic placement of *C.punctatonuchalis* and *C.vandeventeri*. PeerJ 5: e2884. 10.7717/peerj.2884 PMC5267628 28149678

[B42] Wood Jr PL Quah ES Anuar S Muin MA (2013) A new species of lowland karst dwelling *Cnemaspis* Strauch 1887 (Squamata: Gekkonidae) from northwestern Peninsular Malaysia. Zootaxa 3691(5): 538–558. 10.11646/zootaxa.3691.5.2 26167602

[B43] Woodruff DS (2010) Biogeography and conservation in Southeast Asia: how 2.7 million years of repeated environmental fluctuations affect today’s patterns and the future of the remaining refugial-phase biodiversity. Biodiversity and Conservation 19: 919–941. 10.1007/s10531-010-9783-3

[B44] Woodruff DS Turner LM (2009) The Indochinese-Sundaic zoogeographic transition: a description and analysis of terrestrial mammal species distributions. Journal of Biogeography 36(5): 803–821. 10.1111/j.1365-2699.2008.02071.x

